# Which features of ambulatory healthcare are preferred by people aged 80 and over? Findings from a systematic review of qualitative studies and appraisal of confidence using GRADE-CERQual

**DOI:** 10.1186/s12877-022-03006-6

**Published:** 2022-05-16

**Authors:** Angélique Herrler, Helena Kukla, Vera Vennedey, Stephanie Stock

**Affiliations:** 1 Faculty of Human Sciences and Faculty of Medicine, Graduate School GROW – Gerontological Research on Well-being, University of Cologne, Albertus-Magnus-Platz, 50923 Cologne, Germany; 2 Institute for Health Economics and Clinical Epidemiology, University Hospital Cologne, 50935 Cologne, Germany

**Keywords:** Aged, 80 and over, Patient-centered care, Ambulatory care, Qualitative research, Patient preferences, Systematic review

## Abstract

**Background:**

Despite healthcare providers’ goal of patient-centeredness, current models for the ambulatory (i.e., outpatient) care of older people have not as yet systematically incorporated their views. Moreover, there is no systematic overview of the preferable features of ambulatory care from the perspective of people aged 80 and over. Therefore, the aim of this study was to summarize their specific wishes and preferences regarding ambulatory care from qualitative studies.

**Methods:**

The study was based on qualitative studies identified in a prior systematic review. Firstly, the findings of the qualitative studies were meta-summarized, following Sandelowski and Barroso. Secondly, a list of preferred features of care from the perspective of older people was derived from the included studies’ findings through inductive coding. Thirdly, the review findings were appraised using the GRADE-CERQual tool to determine the level of confidence in the qualitative evidence. The appraisal comprised four domains: methodological limitations, coherence, data adequacy, and data relevance. Two reviewers independently evaluated every review finding in each domain. The final appraisals were discussed and ultimately summarized for the respective review finding (high, moderate, low, or very low confidence).

**Results:**

The 22 qualitative studies included in the systematic review were mainly conducted in Northern and Western Europe (*n* = 15). In total, the studies comprised a sample of 330 participants (*n* = 5 to *n* = 42) with a mean or median age of 80 and over. From the studies’ findings, 23 preferred features of ambulatory care were identified. Eight features concerned care relationships (e.g., “Older people wish to receive personal attention”), and 15 features concerned healthcare structures (e.g., “Older want more time for their care”). The findings emphasized that older people wish to build strong relationships with their care providers. The majority of the review findings reached a moderate or high confidence appraisal.

**Conclusions:**

While the listed features of healthcare structures are common elements of care models for older people (e.g., Geriatric Care Model), aspects of care relationships are somewhat underrepresented or are not addressed explicitly at all. Future research should further explore the identified preferred features and their impact on patient and care outcomes.

**Supplementary Information:**

The online version contains supplementary material available at 10.1186/s12877-022-03006-6.

## Background

The population of people aged 80 and over is the fastest growing age group worldwide [1]. It is frequently said that healthcare systems are not adequately prepared for this demographic change [2–5]. The biggest challenge described in most research and policy papers is the increasing burden of disease due to chronic diseases, multimorbidity, frailty and disability [2, 4, 6–9]. However, most healthcare systems are still characterized by fragmented care and an orientation toward acute care [4, 10–12]. It is argued that these preconditions result in inadequate treatments and deteriorations in patient health, and therefore lead to more frequent use of services and rising costs [2, 4, 9, 13]. Although the use of services and rising costs not only affect ambulatory (i.e., outpatient) care, this area has a particular importance since ageing in place and the prevention of institutionalisation are important personal and political goals [5].

As a result, several concepts and models, especially for ambulatory healthcare, have been developed. The most popular approaches and propositions are integrated care and improved sharing of information, interdisciplinary teamwork, the redesign of healthcare workers’ roles, and coordinated care and case management, as well as (geriatric) assessments [2, 3, 7, 8, 10, 12, 14–17]. Moreover, the empowerment of the patient, and the discussion of needs and goals, as well as prevention and health promotion, are required [2, 3, 12]. Most ambulatory care models designed for older people therefore incorporate these aspects (e. g., Geriatric Care Model [18], GRACE [19], Guided Care Model [20], Embrace [21]).

While patient empowerment and autonomy are now frequently described goals of these models, it is surprising that most did not systematically incorporate older patients themselves in their development. Therefore, the features chosen mainly represent the professional (medical) perspective and not necessarily what is important to older people. This applies particularly to those aged 80 and over, since the common models mainly address age groups starting at around 65 years. By contrast, to achieve patient-centred care, which has been claimed as the overarching aim in the design of healthcare, the values, preferences and needs of the patients should be evaluated and put centre stage [5, 22].

In a recent systematic review and meta-synthesis of 22 qualitative studies, we identified three basic needs of people aged 80 and over regarding ambulatory care: *feeling safe*; *feeling like a meaningful human being*; and *maintaining control and independence* [23]. While these findings explain older people’s general view of ambulatory healthcare, the studies also provide several direct descriptions of specific features which could have tangible implications for practice and the design of healthcare. However, there has been no systematic overview of the genuine perspectives of people aged 80 and over regarding the preferable features of the design of ambulatory care. Therefore, we aimed to re-analyze the findings of the primary studies in order to answer the question: *What are the specific preferences and wishes of older people regarding favorable aspects of ambulatory healthcare?* By “preferences,” we mean “what patients want from their healthcare” ([24], p. 168).

## Methods

### Data basis

We conducted a secondary analysis and appraisal of confidence in review findings based on qualitative studies from a previous systematic review of the question: *What matters to older people regarding their ambulatory care?* [23]. The review incorporated a comprehensive database search in Web of Science Core Collection, Medline, PsycINFO and CINAHL, which was complemented by a keyword search in Google Scholar, as well as by a forward and backward citation search. Qualitative primary study reports exploring the subjective preferences, wishes, needs and experiences of people aged 80 and over in ambulatory healthcare settings, performed by professionals, were included. The search covered full research reports published in English, Dutch and German from inception to October 2020, and led to 5576 potentially relevant research reports. Their titles, abstracts and full texts were screened against the eligibility criteria by two independent reviewers, who agreed to include 23 research reports. After independently appraising the quality of the studies, 22 reports were finally included. Further details on the search and selection process are reported elsewhere [23]. Reporting in this paper is based on the recommendations of Tong et al. for qualitative syntheses (ENTREQ) [25].

### Analysis

While the aim of the original review was to integrate the findings of the primary studies and, consequently, provide a new interpretation of them, the present analysis focuses on the extraction and condensation of specific preferable features of healthcare. Moreover, we were interested in the features’ trustworthiness for use in evidence-based practice. Therefore, we started with an aggregation of the 22 studies identified using the meta-summary approach. According to Sandelowski and Barroso, a meta-summary comprises several steps: extracting data, clustering data and abstracting findings [26]. Firstly, the qualitative results that explicitly referred to the older people’s perspective were extracted (mainly from the “findings” section of the study reports) and transferred to MAXQDA Analytics Pro 2020 for analysis (Verbi software, Berlin). Secondly, two authors (AH, HK) independently coded the findings of the studies line-by-line, clustered them around different aspects of healthcare (e.g., “access”), and discussed their results until a consensus was reached. Thirdly, the first author condensed the contents of the clusters around specific features of ambulatory care. The results were discussed by the research team. Their extent was iteratively refined, and clear statements on the desirability of the care feature (e.g., “older people accept/reject/prefer (…)”) were formulated.

### Appraisal of confidence in the evidence

In the subsequent step, the review findings were examined in terms of their informative value and trustworthiness. In the past, this was often done by calculating the frequencies of the qualitative findings [26]. Since this approach follows a quantitative logic and is therefore not ideal for qualitative research, we decided to conduct a detailed confidence appraisal using the relatively new GRADE CERQual tool (“Confidence in the Evidence from Reviews of Qualitative Research”) [27, 28]. CERQual is used to appraise each review finding in four different domains: methodological limitations; coherence; data adequacy; and data relevance [28]. The assessment of methodological limitations was based on the quality appraisal of each included study using the qualitative studies checklist issued by the National Institute for Health and Care Excellence [29]. For each review finding, the quality appraisals of the studies contributing to the respective finding were afterwards summarized and checked for important limitations, e.g., in study design or data analysis [30] (Additional file 1). Coherence was assessed as the fit between the original data and the resulting review finding [31]. Each review finding was compared with all original text segments that it was based on and with the themes developed in the contributing studies. Contradictory data, alternative descriptions or explanations, and unclear data support were considered for rating coherence [31] (Additional file 2). Data adequacy was assessed similarly, but with a focus on data richness and quantity in order to explore the validity and explanatory power of the respective review finding [32] (Additional file 2). Data relevance was assessed as the fit between the review question and the context of included studies. For each review finding, the studies contributing to it were checked for the population, setting and phenomenon of interest (Additional file 3). Two authors (AH, HK) independently assessed each domain per review finding and rated it (*no or very minor concerns*; *minor concerns*; *moderate concerns*; or *serious concerns*). The ratings were discussed until a consensus was reached; a third author (VV) was consulted, where necessary.

Finally, a CERQual qualitative evidence profile for every review finding evolved in which each received an appraisal for every domain*.* The final appraisals of the four domains were discussed by the research team and ultimately summarized for the respective review finding [28]:

- “*High confidence*: It is highly likely that the review finding is a reasonable representation of the phenomenon of interest.
- *Moderate confidence*: It is likely that the review finding is a reasonable representation of the phenomenon of interest.
- *Low confidence*: It is possible that the review finding is a reasonable representation of the phenomenon of interest.
- *Very low confidence*: It is not clear whether the review finding is a reasonable representation of the phenomenon of interest” [27, p. 6].

## Results

The 22 qualitative studies that served as a basis were mainly conducted in Northern and Western Europe and in total comprised 330 participants who lived at home. The participants were mainly multimorbid or frail, and showed a broad range of chronic conditions. The care settings examined were 1) general or specialist ambulatory healthcare (nine studies), 2) home care/community-based long-term care (seven studies), 3) case management (three studies) and 4) home visits (three studies). There were three studies that conducted focus groups or group interviews, all the other studies conducted individual or couple interviews. An overview on the studies is provided in Table 1.

**Table 1:** Overview of studies

**Study**	**Aim**	**Inclusion criteria/Sample**	**Setting**^a^	**Themes**
Behm et al. 2013 [33] Sweden	Description of older people's experiences of a preventive home visit and meaning for (future) health	*N* = 17 participants aged 80 or older who live at home, are cognitively intact and are independent of help, recruited via “Elderly persons in the risk zone” intervention (seven men, twelve women)	Home visits	• The preventive home visit (PHV) made me visible and proved my human value • The PHV brought a feeling of security • The PHV gave an incentive to action • The PHV was not for me
Berkelmans et al. 2010 [34] Netherlands	Description of non-medical service and product attributes older people value in GP care	*N* = 13 participants (mean age 81.2 years), recruited via four GP practices (six men, seven women)	Ambulatory general practice or specialist care	• Continuity of caregiver • Distance to the practice • Accessibility • Expertise and trust • Attitude • Information • Pro-active Initiatives • Waiting time in the waiting room • Free choice of GP
Bjornsdottir 2018 [35] Iceland	Understanding of the nature of home care nursing practice	*N* = 15 home care nursing clients aged 80 or over, identified as frail, recruited via home care nurses (six men, nine women)	Home care and community-based long-term care	• The world at home • Relating to an ailing body and treatments • Give-and-take – life in relations • Home care services as world making
Faeo et al. 2020 [36] Norway	Description of experiences and attitudes of home-dwelling persons with dementia regarding assistive technology, volunteer support, home care services and day care centers	*N* = 12 participants aged 65 and over (mean age 82 years) with a registered dementia diagnosis who live at home, recruited via four daycare centers (six men, six women)	Ambulatory general practice or specialist care	• (Assistive technology – safety with side effects) • (Volunteer support – the complexity of preferences) • Home care services – the diversity of car experience • Daycare centers – it’s all in the details
Gowing et al. 2016 [37] UK	Exploration of views and experiences of patients and carers regarding a case management programme	*N* = 16 older participants (median age 82.5 years) enrolled in the Northumberland High Risk Patient Programme who live at home and do not receive palliative care, recruited via 11 GP practices (five men, eleven women) (*N* = 7 family members)	Case management	• Awareness and understanding of the NHRPP • Confidence in the primary healthcare team • Limitations of home care • The active role of being a patient
Jarling et al. 2017 Sweden	Description of meaning of home care from the perspective of multimorbid older people	*N* = 12 home care clients aged 75 or older (77–90 years) with multimorbidity who live alone, recruited via the municipality’s contact person (four men, eight women)	Home care and community-based long-term care	• Becoming a guest in your own home • Adapting to a caring culture • Feeling exposed • Unable to influence care • Forced relations
King et al. 2017 New Zealand	Description of experiences of older people and health professionals regarding a primary healthcare gerontology nurse specialist role	*N* = 5 participants aged 75 and older (mean age > 80 years) who received the intervention, recruited via primary healthcare practices (selection from a random numbers table; two men, three women) (*N* = 6 healthcare professionals)	Case management	• Holistic expertise • Communication • (Competency) • (Service delivery)
Krothe 1992 [38] USA	Description of community-based services needed by older people to avoid institutionalization	*N* = 9 clients of an Area Agency on Ageing (mean age 81.4 years), recruited via the agency’s case manager (two men, seven women)	Home care and community-based long-term care	• Maintaining control • Goal setting • The nursing home • Role of family • Essential formal services • Informal help/assistive devices • Significance of home and possessions • Day to day activities/community connectedness • Finding out about CB-LTC • Future needs for CB-LTC and assisted living • Significance of past experience • Loss theme • Spirituality • Listening for individualized needs • Some elderly people are like that • Being alone and loneliness
Martin-Matthews & Sims-Gould 2008 [39] Canada	Description of salient home support services issues from the perspective of employers, home support workers and clients	*N* = 14 home care clients (mean age 83 years), recruited via home support agencies (four men, ten women) (*N* = 11 home care employers and *n* = 32 home support workers)	Home care and community-based long-term care	• (Recruitment and retention) • (Increasing complexity of client needs) • (Acknowledgement of the needs and desires of clients) • (Appropriateness of home support as part of the healthcare continuum) • (Scheduling and time demand) • (Tension in providing intimate ongoing care at an emotional distance) • (Balance between tasks outlined in the care plan and the needs and wants of elderly clients) • Ongoing need to prepare for and manage service • Desire and need for companionship
Michel et al. 2015 [40] Brazil	Analysis of similarities and dissimilarities in the meanings assigned to healthcare by older people and nursing professionals	*N* = 10 participants aged 80 and over who were users of the basic health unit for at least six months (five men, five women) (*N* = 10 nursing professionals)	Ambulatory general practice or specialist care	• “Because we are older”: reasons to provide health care to long-lived elders • “Being well served” and more help at home: attributes of health care for long-lived elders • Health services and practices that do good: used to provide health care to long-lived elders • (Old age and vulnerability: reasons to provide health care to long-lived elders) • (Deficits in proper care: attributes of health care for long-lives elders) • (Responsibility of families and guidance: used to provide health care to long-lives elders)
Modig et al. 2012 [41] Sweden	Description of frail older people's experiences regarding information about their medications	*N* = 12 participants aged 65 and older (median age 80.5 years), needing help with two or more ADL, who were admitted to hospital twice or more, had at least four outpatient contacts in the prior twelve months, and received cardiovascular medication; recruited via a case manager intervention study (five men, seven women)	Ambulatory general practice or specialist care	• Comfortable with information • Insecure with information
Moe et al. 2013 [42] Norway	Description of the meaning of receiving home nursing care for chronically ill older people living at home	*N* = 11 participants aged 80 and over, living at home with chronic conditions, and receiving home nursing care (five men, six women)	Home care and community-based long-term care	• Being ill and dependent on help • Being at the mercy of help • Feeling inferior as human being
Sandberg et al. 2014 [43] Sweden	Description of frail older people's and case manager's experiences of a case management intervention	*N* = 14 participants aged 65 or older (mean age 83 years) who received the intervention, lived in an ordinary home, needed help for at least two ADL, were admitted to hospital at least twice and had for outpatient care contacts in the prior twelve months; recruited via one university hospital, four primary care centers, the municipal home care organization (four men, ten women) (*N* = 6 case managers)	Case management	• The case manager as a helping hand • Case management as a possible additional resource • (The case manager as a coaching guard) • (Case management as entering a new professional role)
Schulman-Green et al. 2006 [44] USA	Description of older adults’ interaction regarding their life and health goals during the clinical encounter	*N* = 42 participants aged 60 or over (mean age 81 years) living in a high-income independent living facility, a subsidized assisted living facility or a private condominium complex, identified by a contact person for each residential site (15 men, 25 women) (*N* = 11 clinicians)	Ambulatory general practice or specialist care	• Not a priority given limited time • Focus on symptoms • Clinician-patient mutual perception of disinterest in goal setting • Presumption that all patients’ goals are the same
Soodeen et al. 2007 [45] Canada	Description of home care experiences of physically impaired older people and their spouses	*N* = 9 home care receivers (mean age 80 years) with at least one ADL or IADL and one chronic condition, recruited via newspaper a article, referrals from seniors housing complexes and the staff of church-run programs (three men, six women) (*N* = 9 spouses)	Home care and community-based long-term care	• Independence • Developing a trusting relationship with home care workers • (Relief) • (Continuity)
Spoorenberg et al. 2015 [46] Netherlands	Description of older adults’ perspective regarding integrated care and support	*N* = 23 participants of the intervention (mean age 82 years); frail people or those with complex care needs were recruited by their case managers; robust people were recruited by project managers (ten men, 13 women)	Ambulatory general practice or specialist care	• Experiences with aging ◦ Struggling with health ◦ Increasing dependency ◦ Decreasing social interaction ◦ Loss of control ◦ Fears • Experiences with Embrace ◦ Relationship with the case manager ◦ Interactions ◦ Feeling in control, safe and secure
Tiilikainen et al. 2019 [47] Finland	Description of older people's perceptions of quality of life from the perspective of access and use of health and social care services	*N* = 19 participants who lived alone (mean age 80 years) and received health and social services during the past six months, recruited via local health and social service professionals (four men, 15 women)	Ambulatory general practice or specialist care	• Access to services and information • Recognition inside the services
Toien et al. 2015 [48] Norway	Description of older people's perspectives regarding preventive home visits	*N* = 10 participants (mean age 85.5 years) who had at least six years of experience with the preventive home visits service and with various characteristics, recruited via a municipal health care service nurse (four men, 6 women)	Home visits	• To feel safe • To manage daily life • To live well • To be somebody
Turjamaa et al. 2014 [49] Finland	Description of older people's and practical nurses' perspectives regarding available home care and enablers for continuity of living home	*N* = 23 home care clients aged 75 or older (mean age 84 years) with at least one or two home visits a day, recruited via practical nurses (*N* = 14 practical nurses)	Home care and community-based long-term care	• Organisationally driven care • Individual encountering the multifaceted system
van Blijswijk et al. 2018 [50] Netherlands	Description of older people's experiences regarding hindering health complaints, how they deal with them and what they expect from their GP	*N* = 24 participants aged 80 or older with pain and/or problems with walking/standing, recruited via an integrated care trial (six men, 18 women)	Ambulatory general practice or specialist care	• Health complaints and impact • Self-management of health complaints and limitations • Expectations of their GP concerning their health complaints ◦ Shared decision-making ◦ Pro-active care ◦ Attentive care: support and empathy ◦ Attainability and accessibility ◦ Coordinating health care and medication
van Kempen et al. 2012 [51] Netherlands	Description of frail older people's views and needs regarding home visits	*N* = 11 frail patients aged 65 or over (median age 80 years, two men, nine women) (*N* = 11 informal caregivers)	Home visits	• The need for home visits • Preferences for home visits
Walker et al. 2018 [52] Australia	Description of older dementia patients' and their family caregivers' experiences and preferences regarding dementia assessment services	N = 9 participants aged 65 or older (mean age 80 years) with a formal diagnosis of mild dementia within the prior three months, recruited via a geriatrics service and an Alzheimer’s Association (five men, four women) (*N* = 7 caregivers)	Ambulatory general practice or specialist care	• Being “handled” properly: facilitators and barriers to a formal diagnosis • Perceptions on length of time between diagnosis and accessing support services • Preferences for diagnostic service settings: importance of avoiding stigma

In some studies, additional participant groups, such as caregivers were included and some of the primary studies’ results apply only to them. In our analysis, we included only findings that explicitly referred to our target group. However, other groups and results of the primary studies are reported in parentheses to enhance transparency

^a^The studies were assigned to four different contexts: 1) ambulatory general or specialist healthcare, 2) home care/community based long-term care, 3) case management, 4) home visits

*GP* General practitioner/practice, *ADL* Activities of daily living, *IADL* Instrumental activities of daily living, *CB-LTC* Community-based long-term care

**Fig. 1:**
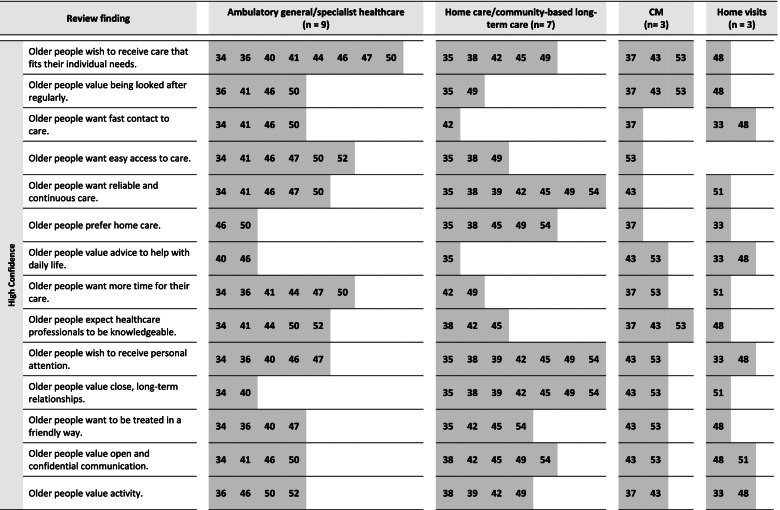
Review findings with high appraisal of confidence and care contexts of the contributing studies. Note: The numbers in the row are the references of the studies contributing to the respective review finding, sorted by their care contexts. n, total number of studies included from the respective care context

**Fig. 2:**
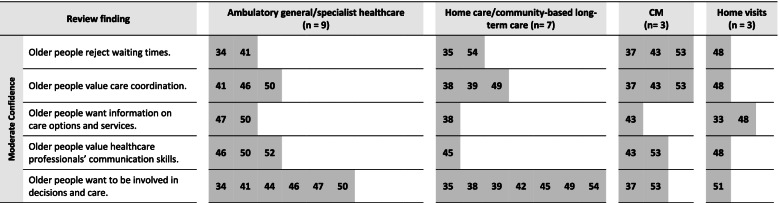
Review findings with moderate appraisal of confidence and care contexts of the contributing studies. Note: The numbers in the row are the references of the studies contributing to the respective review finding, sorted by their care contexts. n, total number of studies included from the respective care context

**Fig. 3:**
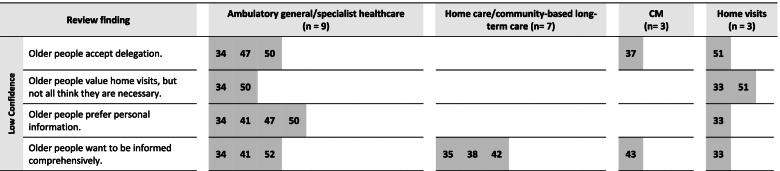
Review findings with low appraisal of confidence and care contexts of the contributing studies. Note: The numbers in the row are the references of the studies contributing to the respective review finding, sorted by their care contexts. n, total number of studies included from the respective care context

From these 22 studies, our analysis resulted in 23 review findings on the preferable features of ambulatory care. The majority of them reached moderate or high confidence. Fifteen review findings concerned the structures of healthcare and eight review findings concerned care relationships. In the following section, each review finding will be presented with a short description to provide a better understanding of its meaning. Table 2 summarizes the findings, together with their overall CERQual rating and examples for supporting data. The CERQual qualitative evidence profile (Additional file 4) provides an overview of the assessments and explanations for each appraisal domain. To provide a better understanding of the findings’ applicability, Figs. 1, 2 and 3 show the review findings in relation to the examined care settings of the contributing studies.

**Table 2:** Summary of qualitative findings and CERQual assessments of confidence

**Summary of review finding**	**Contributing studies**	**Example for data support** (original citations from the qualitative studies’ participants)	**CERQual assessment of confidence**	**Explanation of CERQual assessment**
Features of healthcare structures				
1. Older people wish to receive care that fits their individual needs	[34–38, 40–50, 53]	“It is what they do – they who are the right persons… they do something extra. They have learned to treat us as we want” ([42], p.742)	High	Seventeen studies with no or very minor concerns regarding methodological limitations and adequacy contributed to this review finding. Although there were minor concerns about coherence and relevance, this was only due to a limited number of studies/extent of data
2. Older people value being looked after regularly	[35–37, 41, 43, 46, 48–50, 53]	“The most important is the safety – you know, that someone cares and looks after you and checks that the head is still functioning; that is very reassuring. And knowing you are within the municipality’s system” ([48], p. 704)	High	Ten studies with no or very minor concerns regarding adequacy and relevance contributed to this review finding. Although there were minor concerns about methodological limitations and coherence, this was only due to a limited number of studies/extent of data
3. Older people accept delegation	[34, 37, 47, 50, 51]	“Or he’ll send the head nurse… to see what’s the matter. One of them would be here and see exactly what’s the matter and she would confer with him [the GP] what was to be done” ([37], p. 4)	Low	Five studies contributed to this review finding. While there were no or very minor concerns regarding methodological limitations, there were moderate concerns regarding coherence and adequacy because of the small number of studies and partially contradictory data. Moreover, there were minor concerns about relevance
4. Older people value home visits, but not all think they are necessary	[33, 34, 50, 51]	“The GP can go through his patient records to see which patients need a home visit, which patients really need it” ([51], p. e557)	Low	Four studies contributed to this review finding. While there were no or very minor concerns regarding methodological limitations and relevance, there were moderate concerns regarding coherence and adequacy because of the small number of studies and partially contradictory data
5. Older people want fast contact to care	[33, 34, 37, 41, 42, 46, 48, 50]	“I know who to call, and I am certain that I will get help the day I need. It cannot be any better” ([48], p. 704)	High	Eight studies contributed to this review finding. There were no or very minor concerns regarding methodological limitations, coherence, adequacy and relevance
6. Older people want easy access to care	[34, 35, 38, 41, 46, 47, 49, 50, 52, 53]	“It goes through so many different levels before you actually get any help […]. If you need them, they’re not there” ([46], p. 9)	High	Ten studies contributed to this review finding. There were no or very minor concerns regarding methodological limitations, coherence and adequacy. Although there were moderate concerns regarding relevance, the review finding still is a valid representation of the data
7. Older people reject waiting times	[34, 35, 41, 54]	“I come here for an appointment and wait for three hours. There is no single time I have come here when my blood pressure hasn’t gotten higher, I guess I get angry. Where is the priority on old age? At least above 80 years old. I’m 87” ([40] p. 346)	Moderate	Four studies contributed to this review finding. There were no or very minor concerns regarding methodological limitations and coherence. However, there were minor concerns regarding adequacy and relevance and due to the quite small number of studies, we found that this weakened the review finding
8. Older people want reliable and continuous care	[34, 35, 38, 39, 41–43, 45–47, 49–51, 54]	“Never the same [nurse]. Do not know how many different persons they are? I do not know who is coming you know” ([42], p. 740)	High	Fourteen studies contributed to this review finding. There were no or very minor concerns regarding methodological limitations, coherence and adequacy. Although there were minor concerns regarding relevance, there was in sum no negative impact on the review finding
9. Older people value care coordination	[37–39, 41, 43, 46, 48–50, 53]	“She was wonderful, she was a wonderful help… she sorted my doctor out, and sorted my nurse out” ([53], p. 811)	Moderate	Ten studies contributed to this review finding. There were no or very minor concerns regarding coherence and adequacy. However, there were moderate concerns regarding methodological limitations and relevance that weakened the review finding in total
10. Older people prefer home care	[33, 35, 37, 38, 45, 46, 49, 50, 54]	“You feel best at home, this is your home, where your things are. The home is part of you. Being at home means that everything is friendly and free” ([35], p. 3)	High	Nine studies contributed to this review finding. There were no or very minor concerns regarding methodological limitations and adequacy. Although there were minor concerns regarding coherence and relevance, this did not significantly affect the review finding, which was still a valid representation of the data
11. Older people prefer personal information	[33, 34, 41, 47, 50]	“Well, I think you absorb better, you understand it better, what’s available. Otherwise I think that we would just have thrown away the brochures and thought that we would wait to deal with it until something happens. Now we know about this, we have received a visit, it remains in our memory” ([33], p. 5)	Low	Five studies contributed to this review finding. While there were no or very minor concerns regarding methodological limitations, there were moderate concerns regarding relevance. Moreover, there were minor concerns regarding coherence and adequacy. Since the number of contributing studies was small, we found that this significantly impacted the strength of the review finding
12. Older people value advice to help with daily life	[33, 35, 40, 43, 46, 48, 53]	“And I find it very difficult to keep my balance. And they [name, physiotherapist in the project] asked me how would it be if you stood with your legs further apart… then your balance will be a bit better… And I’ve been doing it, and it’s absolutely true, because now I can stand and wash up” ([43], p. 9)	High	Seven studies contributed to this review finding. There were no or very minor concerns regarding coherence, adequacy and relevance. However, there were moderate concerns regarding methodological limitations. Since this is mostly due to one study, there was no significant impact on the review finding in total
13. Older people want information on care options and services	[33, 38, 43, 47, 48, 50]	“She understands my problems and has suggested a number of assistive devices that I neither knew existed nor knew that I could get. I could not have managed without those helping aids. Thanks to them, I can now live close to normal” ([48], p. 705)	Moderate	Six studies contributed to this review finding. While there were no or very minor concerns regarding methodological limitations, there were minor concerns regarding coherence and adequacy. Moreover, there were moderate concerns regarding relevance that weakened the review finding
14. Older people want to be informed comprehensively	[33–35, 38, 41–43, 52]	“When I get a new pill, she usually goes through it with me; she usually says what it is good for and how it works and such things. And I should watch if I have something more than what is written in the leaflet. If something else happens” ([41], p. 5)	Low	Eight studies contributed to this review finding. There were no or very minor concerns regarding methodological limitations. However, there were moderate concerns regarding coherence, adequacy and relevance. Since there was one study with limitations that provided a large part of data and moreover, there were partially contradictory data, there was a strong weakening of the review finding
15. Older people want more time for their care	[34, 36, 37, 41, 42, 44, 47, 49–51, 53]	“I just wish the GP would listen to me for a while. Just sit there and listen to me and give me my say…. I think just let me try and explain things to you. But he’s a very busy man” ([53], p. 812)	High	Thirteen studies contributed to this review finding. There were no or very minor concerns regarding methodological limitations, coherence, adequacy and relevance. Although there were minor concerns regarding relevance, this did not impact the strength of the review finding
Features of care relationships				
16. Older people expect healthcare professionals to be knowledgeable	[34, 37, 38, 41–45, 48, 50, 52, 53]	“I also expect him to keep his level of knowledge up to par with his skills. By which I mean, that he takes refresher courses regularly” ([34], p. 4)	High	Twelve studies contributed to this review finding. There were no or very minor concerns regarding methodological limitations, coherence and adequacy. Although there were moderate concerns regarding relevance, the review finding was still a valid representation of the data
17. Older people value healthcare professionals' communication skills	[43, 45, 46, 48, 50, 52, 53]	“She explained everything so well… that made a difference” ([53], p. 810)	Moderate	Seven studies contributed to this review finding. There were no or very minor concerns regarding coherence and relevance. However, there were minor concerns regarding adequacy and moderate concerns regarding methodological limitations. Altogether, we found that this weakened the review finding, but to a limited extent
18. Older people wish to receive personal attention	[33–36, 38–40, 42, 43, 45–49, 53, 54]	“Just that they think about us, it's nice, they think of older people” ([33], p. 4)	High	Sixteen studies contributed to this review finding. There were no or very minor concerns regarding methodological limitations, coherence, adequacy and relevance
19. Older people value close, long-term relationships	[34, 35, 38–40, 42, 43, 45, 49, 51, 53, 54]	“They have become my friends, and I can rely on them” ([35], p. 5)	High	Twelve studies contributed to this review finding. There were no or very minor concerns regarding coherence, adequacy and relevance. Although there were minor concerns regarding methodological limitations, this was only due to a small number of studies and there was no impact on the review finding in total
20. Older people want to be treated in a friendly way	[34–36, 40, 42, 43, 45, 47, 48, 53, 54]	“I want them to be honest and also I want them to be friendly” ([45], p. 1249)	High	Eleven studies contributed to this review finding. There were no or very minor concerns regarding methodological limitations, coherence and adequacy. Although there were minor concerns regarding relevance, this was only due to a small number of studies and there was no impact on the review finding in total
21. Older people value open and confidential communication	[34, 38, 41–43, 45, 46, 48–51, 53, 54]	“And you could talk to her… about everything. About things I do not want to mention to you. But I developed very good trust to her” ([43], p. 9)	High	Thirteen studies contributed to this review finding. There were no or very minor concerns regarding methodological limitations, coherence, adequacy and relevance
22. Older people want to be involved in decisions and care	[34, 35, 37–39, 41, 42, 44–47, 49–51, 53, 54]	“Once I had an infection in my wrist and that was solved—but he’s never asked about it again. That’s a little bit of response you would like to receive, that you feel that we’ve solved the problem together” ([50], p. 9)	Moderate	Sixteen studies contributed to this review finding. There were no or minor concerns regarding adequacy, but minor concerns regarding methodological limitations, coherence and relevance. In total, we found that the review finding lost strength, in particular due to contradictory data
23. Older people value activity	[33, 36–39, 42, 43, 46, 48–50, 52]	“Now you are old, but look how much you can do, and it’s me who will do it. It’s not them, it’s me who will do all the things they talked about. I need to engage in all these activities, I cannot just sit. … I have an insight, an insight into everything that I can do now and that feels very important” ([33], p. 5)	High	Twelve studies contributed to this review finding. There were no or very minor concerns regarding methodological limitations, coherence, adequacy and relevance

### Review findings with high appraisal of confidence

#### Older people wish to receive care that fits their individual needs

For older people, one remarkable feature was that they wanted to receive holistic care, sufficient support that met their needs and supported their independence, and individual adjustments to care and care planning [34–38, 40–43, 45, 46, 48–50, 53]. Concomitantly, older people negatively judged experiences such as not receiving the care needed (regarding lack of time, money, personnel or individual attention), not receiving individual adjustments to care, not been taken seriously with their concerns, and a focus on acute problems and symptoms instead of a long-term perspective and goal setting [35, 37, 38, 40–42, 44, 45, 47, 50].

#### Older people value being looked after regularly

The older people highly appreciated having a healthcare professional who “kept an eye” on them. They felt reassured when someone monitored their health status, looked after them regularly, cared for chronic issues (e.g., wounds, medications) and could intervene fast, if necessary [35–37, 41, 43, 46, 48–50, 53]. However, a specific frequency or contact interval was not proposed.

#### Older people want fast contact to care

It became apparent that older people wish to make contact with a (known) healthcare professional fast, if needed. They prefer to have a constant person or healthcare practice which they could contact if advice or help was required [33, 34, 42, 46, 48]. Widespread and fast availability via phone was especially valued [34, 37, 41, 42, 48, 50].

#### Older people want easy access to care

Most participants in the qualitative studies had already experienced difficulties or restrictions in accessing healthcare, e.g., for specialist services. Although some of them mentioned that the proximity of services was good, widespread access to healthcare, including on weekends and on an intermittent basis, as well as easily accessible follow-up services and referrals, were rated most important [34, 35, 38, 41, 46, 47, 50, 52]. Older people identified restricted opening hours, the fragmented nature of the care systems, and the need to go through several levels of care before receiving the right treatment as barriers to good access [38, 46, 47, 49, 50, 52].

#### Older people want reliable and continuous care

Older people frequently reported a high turnover of healthcare professionals responsible for them but strongly desired continuity. They wanted their caregivers to know them personally and to have a good overview of their living circumstances and care needs [34, 37–39, 41–43, 45–47, 49–51, 54]. A lack of continuity led to stress, unstructured and impersonal care, insecurity and information loss [38, 39, 41, 42, 49, 54]. Furthermore, they wanted reliability in receiving care, e.g., a regular schedule and predictability [35, 39, 48, 54].

#### Older people prefer home care

Consistently, study participants expressed a strong desire to stay in their own homes for as long as possible because of the better quality of life, increased privacy and control, and the belief that their homes offered a more secure environment [33, 35, 37, 38, 45, 46, 50, 54]. They acknowledged that receiving home care and support was needed for them to age in place [35, 38, 45, 46, 49]. There were indications that, in contrast, a nursing home would constitute a threat to older people’s personal integrity and quality of life; they had quite a negative view of institutional care [38, 46]. Seldom was institutional care perceived as the better option to meet their needs [37].

#### Older people value advice to help with daily life

Several studies found that older people value receiving advice to help with their daily lives. They welcomed practical advice for adaptations of their home (e.g., the removal of carpets to prevent falls), safety information and education regarding health issue prevention and diet, and recommendations for exercises [33, 35, 40, 43, 46, 48, 53].

#### Older people want more time for their care

A major obstacle to favorable care was time constraints. Participants described that their care or medical appointments were frequently rushed and that there was not enough time for the necessary help and conversation [34, 36, 37, 41, 42, 44, 47, 49–51, 53]. Insufficient time for care was described as resulting in unresolved questions and a focus on acute tasks and symptoms, rather than on considering long-term plans and goals [36, 37, 41, 44, 47].

#### Older people expect healthcare professionals to be knowledgeable

The older people expected healthcare professionals to have a certain level of knowledge and experience in order to provide good care, which was also described as a condition for trust [34, 37, 38, 41–45, 48, 50, 52, 53].

#### Older people wish to receive personal attention

Descriptions of care as an important social contact point were relevant in almost all studies, but more frequently in those describing home care and home visits. The interviewees appreciated having the feeling that someone was interested and cared about them [33, 34, 38, 40, 46, 53]. In several studies, it was indicated that the social aspects of care – caregivers spending time with them, starting conversations, providing emotional support – were highly valuable for the older persons’ well-being [33, 36, 38, 39, 42, 43, 45–49, 53, 54]. The older people also revealed this wish for beneficial contacts in describing negative experiences, e.g., caregivers visibly hurrying, not talking and not focusing on them, which resulted in negative feelings and a sense of isolation [35, 38, 42, 45–47].

#### Older people value close, long-term relationships

Establishing close, long-term care relationships was an overall present topic, although mainly related to home care professionals (e.g., nurses) or case managers. Older people wanted trustful interactions with well-known healthcare professionals that enabled them to share personal issues and to feel safe and strengthened [34, 35, 38, 40, 42, 43, 45, 49, 51, 53, 54]. Frequently, it was indicated that they developed friendships or family-like relationships [35, 39, 42, 43, 45, 53, 54].

#### Older people want to be treated in a friendly way

Older people valued a kind, open and positive attitude on the part of caregivers and wanted to be treated respectfully [34, 36, 40, 42, 43, 45, 47, 48, 53]. On the other hand, some studies described how older people felt hurt when caregivers were authoritative, disrespectful, impersonal, rude or – in general – lacked empathy [35, 42, 47, 54].

#### Older people value open and confidential communication

Older people would like to communicate with their care providers in an open and confidential manner. The importance of trust, genuine interest and attention to the person’s broader health concerns and living circumstances were stressed, as well as the possibility of discussing everything with the professionals [34, 43, 45, 46, 48–51, 53, 54]. Concomitantly, the studies’ participants described negative experiences, such as professionals not listening to them, not having the chance to speak about personal problems, and feelings of distrust, shame or being a burden, which resulted in inhibited communication [38, 41, 42, 50, 54].

#### Older people value activity

Several participants expressed the wish to remain as active as possible, e.g., regarding physical activity, volunteer work or social activities. They appreciated care professionals who supported them doing so [38, 42, 43, 46, 48, 50]. Furthermore, the older persons found it highly valuable when care professionals motivated them to improve their health and living circumstances, opened up a new, positive perspective of their possibilities and encouraged them to take on active roles [33, 36, 37, 39, 43, 46, 48, 49, 52].

### Review findings with moderate appraisal of confidence

#### Older people reject waiting times

The older participants found waiting times (waiting for telephone contact, waiting for an appointment, waiting at an appointment) generally problematic [34, 35, 40, 41, 54]. Some explained that their issues were urgent and priority should be given to old age; inconvenience, such as hard benches in waiting rooms, was also mentioned in connection with waiting times [34, 40].

#### Older people value care coordination

Older people greatly appreciated care coordination, including in the form of case management. They felt reassured when their care services, treatments, collaboration between different providers and necessary adaptations were organized and managed by a healthcare professional, someone who had an overview and was able to provide them with additional support, where needed [37–39, 41, 43, 46, 48–50, 53].

#### Older people want information on care options and services

The studies’ participants wanted to receive information on care options, services and additional help, in terms of which were suitable and available for them, where they could be accessed and how they could apply for them [33, 38, 43, 47, 48, 50].

#### Older people value healthcare professionals’ communication skills

Older people valued interpersonal and educational skills, e.g., regarding explanations of treatment. Healthcare professionals that were “good communicators” helped improve the understanding of care and affected older people positively, e.g., by lessening anxiety [43, 45, 46, 48, 50, 52, 53].

#### Older people want to be involved in decisions and care

The majority of studies indicated that older persons wanted to be involved in decision-making and planning regarding their healthcare and lifestyle as autonomous and equal partners [35, 37–39, 41, 42, 44–47, 49–51, 53, 54]. This was described as a wish to be asked about needs and priorities, instead of professionals assuming that they knew what these were, and as a wish to be taken seriously [34, 38, 44, 46, 50, 54]. On the other hand, professionals not taking older people’s perspective into account, acting in a paternalistic way and not discussing individual concerns or goals were judged negatively [42, 44, 47, 53]. Nevertheless, the minority of the older people wanted to be rather passive, relied on care professionals and wanted them to provide care and make decisions, e.g., regarding hospital admission [35, 37, 41, 44].

### Review findings with low appraisal of confidence

#### Older people accept delegation

Regarding general care practices and home visits, most older people accepted task delegation to assistants or nurses, or even welcomed it. On the condition that this person provided a continuous contact, knew them well and exchanged information with a GP or specialist, delegation was found to be a good alternative for minor problems or follow-up appointments, and could even mean that more time and attention was provided for the older person [34, 37, 47, 50, 51]. Nevertheless, some older people preferred contact with a physician and sometimes considered nurses and assistants to be barriers to physician access [34, 50, 51].

#### Older people value home visits, but not all think they are necessary

Home visits were discussed controversially in the qualitative studies. In general, it became apparent that receiving a home visit was seen as favorable if someone really needed it but was not required in less urgent cases [34, 51]. Nevertheless, home visits were welcomed as offering the potential for personal attention and as providing more information on the older person’s living circumstances and psychosocial context [34, 50, 51]. By contrast, one study on preventive home visits found that these could be too demanding for some ill people [33].

#### Older people prefer personal information

Older people found it easier to understand information in a face-to-face-conversation, where questions and difficult terms or issues can be discussed directly; brochures or leaflets were requested rather as memory aids [33, 34, 41]. According to the results of two studies, offers of digital services or online communication were refused [47, 50].

#### Older people want to be informed comprehensively

Older people wished to be informed well about their health status, treatments and further issues by healthcare professionals so that they can understand the procedures [33–35, 38, 41–43, 52]. In contrast, it was reported that some did not wish for more explanations and that they were satisfied with limited information [34, 41].

## Discussion

The aim of this study was to summarize the specific preferences and wishes of older people regarding features of ambulatory healthcare. We developed 23 review findings from 22 qualitative studies relating to healthcare structures and care relationships, and appraised the level of confidence in them. Most findings reached a moderate or high confidence level. This was particularly the case for findings that comprised a higher number of contributing studies. Moreover, the inclusion of studies in the systematic review itself was already restrictive regarding characteristics such as the population’s age, resulting in a higher relevance for the findings. Additionally, our findings are of a descriptive nature, so the fit between the findings and the respective contributing data was often direct. However, four of our review findings reached only a low confidence level in the evidence. This was mainly due to a lower number of contributing studies and contrary data. However, a lower confidence rating does not necessarily mean that the findings were unrepresentative. In these cases, further research is especially needed. This also applies to further care settings from which no or only few studies contributed to the presented review findings. So far, these findings should be transferred carefully to other settings – in particular, when between general healthcare and specific aged-care settings.

Many of our findings with a moderate or high level of confidence are in line with other research, e.g., the wish to stay home for as long as possible [55]. However, other findings are more controversial. For instance, our confidence in the finding that older people accept delegation is low and other research on this matter is also ambivalent. A recent representative survey in Germany showed that the majority of adults accept the shifting of medical tasks to medical practice assistants, but the acceptance varied depending on the specific task (in favor of minor illnesses), and adults aged 65 and over tended to be more unwilling [56]. There are also indications that further variables need to be explored to understand older people’s preferences. While our finding that “older people want to be informed comprehensively” received only a low confidence rating due to contrary data, a study on information-seeking preferences among older people (with a mean age of 73 years) found that a lower level of health literacy is associated with a lower desire for information [57]. This is a good example of a feature of care that should be examined in more detail.

A variety of our findings related to aspects of care relationships. This corresponds to other studies exploring the younger age group (65 +) or institutional settings. For instance, Bangerter et al. showed that care providers’ attitude (interest, friendliness, compassion) and communication (active listening, talking) are very important for nursing home residents aged 80 and over, although in urgent cases, fast professional behaviour was preferred [58]. In a population-based survey on the desirable characteristics of professional long-term caregivers, people aged 65 and over especially valued soft skills such as kindness and empathy, and these aspects were much more important than the provider’s gender or ethnical background [59]. In a qualitative study in primary care with people aged 70, Bastiaens et al. also found that good communication skills were valued and that most older patients wanted to have a confidential and caring relationship with their caregivers [60]. Altogether, older people clearly wish to build relationships with care providers and experience empathy.

By contrast, current care models for older people primarily target healthcare structures and the patient’s individual behaviour. When compared to our findings, these models do not fit the subjective needs and preferences of older people. Moreover, it may be possible that this lack of fit affects the success of such models. For instance, some complex care interventions, such as the Geriatric Care Model, did not achieve significant improvements in patients’ quality of life or other outcomes [18]. This may be explained by the fact that despite much criticism, Western countries already provide high-level healthcare structures [18]. While efforts to reform healthcare structures are nonetheless important and often improve clinical outcomes or decrease the use of services [11], addressing care relationships could also be very promising, as our findings show.

In order to complement care for very old people with effective care relationships, it may be helpful to learn from the concept of relationship-centred care. This attempt to humanize and improve care focuses on patients’ relationships and interactions with the care system and their outcomes [61]. Rather than technical communication skills or medical expertise, interpersonal competences are required [62]. Several of our review findings correspond to the elements of relationship-centred care that Dewar and Nolan describe: “willingness to negotiate and compromise, willingness to see another perspective, promoting and accepting the emotions of others, sharing personal information, openness to other ideas, sharing insights when things are not going well, recognizing what people are good at” ([62], p. 1256).

However, the practical reality might look different. One the one hand, primary care providers describe that care for older people is personally and interpersonally challenging [63] and medical students complain about “the emotional burden of caring for older patients” ([64], p. 1996). On the other hand, focusing on relationships rather than on the medical aspects of care may not meet professionals’ expectations and ambitions, and therefore may make caring for older people unattractive [64]. Since older patients are expected to be seen more frequently in most medical subspecialities, apart from geriatrics, addressing attitudes and interpersonal competences in all healthcare professions seems necessary. In the systematic review of Tullo et al. on teaching interventions to improve the knowledge, skills and attitudes of medical students, increased exposure to older patients and long-term teaching implementation were found to be effective [65]. Furthermore, geriatric issues should be presented as “intellectually challenging and emotionally appealing” ([66], p. 241). However, multicomponent interventions in primary care still mainly focus on care structures such as access. Only a few include provider education and training and among these, the content of the training often refers to disease-specific knowledge [67]. Therefore, primary care interventions and innovations do not comprehensively prepare for the growing number of older adults in the population, and seldom address providers’ attitude and care relationships.

Altogether, it becomes clear that besides ambulatory healthcare structures, several features of care relationships are important to people aged 80 and over. While our findings provide an overview of the relevant features of care, future research should further explore these and their impact on relevant patient and care outcomes to enable age-appropriate care. The features of care presented in this paper may serve as a basis for investigations in other (especially non-European) countries and cultures. Moreover, they could provide a basis for quantitative investigations such as discrete choice experiments to strengthen the inclusion of the perspective of people aged 80 and over in the design of healthcare. However, this should not replace discussions about older people’s wishes and preferences in individual care situations.

### Strengths and Limitations

To our knowledge, this is the first systematic overview of the preferable features of ambulatory care from the perspective of people aged 80 and over. The work benefits from a base of 22 studies, which were systematically searched and appraised. Moreover, the rigorous application of CERQual allows for detailed insight into the confidence that can be put in the findings; this therefore strengthens their potential for incorporation into evidence-based decision-making. Since CERQual is a tool designed for qualitative research synthesis, it particularly serves the requirements of qualitative research, instead of referring to frequencies to provide an appraisal of confidence in the evidence. Additionally, the presented approach of analysis and confidence appraisal is particularly suitable to promote the systematic incorporation of qualitative evidence for practice-oriented problems and policy questions (e.g., as in comprehensive health technology assessment reports). Therefore, it complements integrating or theorizing approaches such as meta-ethnography in providing a deeper understanding of, e.g., patients’ perspectives.

However, some limitations should be considered. Firstly, the selection of studies is based on an earlier systematic review and all of its limitations apply here as well: 1) the risk of unconsidered data due to dissemination bias and the restriction to English, German and Dutch publications; 2) restricted transferability to other countries because most of the included studies were conducted in Northern and Western Europe; and 3) restricted transferability to certain care settings, such as dental care, since the studies included did not cover them [23]. Secondly, CERQual is a relatively new tool for appraising qualitative review findings, especially regarding care for older people and their preferences. There may have been pitfalls in the application that we have not registered. In particular, the use of another tool for the appraisal of methodological limitations might have resulted in slightly different confidence ratings.

## Conclusions

This meta-summary provides a set of 23 preferable features of ambulatory care from the perspective of people aged 80 and over. The findings highlight the role of care relationships, which seem to be as yet underrepresented in the design of healthcare. Further research should explore the single features in more detail and their possible effects on patient outcomes and quality of care. The use of qualitative research syntheses in combination with CERQual, as described in this paper, has the potential to allow for systematic inclusion of patients’ perspectives in the design and development of care.

## Supplementary Information

**Additional file 1.** Assessment of methodological limitations.

**Additional file 2.** Assessment of coherence and adequacy.

**Additional file 3.** Assessment of relevance.

**Additional file 4.** CERQual evidence profile.

## Data Availability

The datasets used and analysed during the current study are available from the corresponding author on reasonable request.

## Abbreviation

CERQual: Confidence in the Evidence from Reviews of Qualitative Research
