# Supplementary material for: Which features of ambulatory healthcare are preferred by people aged 80 and over? Findings from a systematic review of qualitative studies and appraisal of confidence using GRADE-CERQual
Source: BMC Geriatr. 2022 May 16;22:428. doi: 10.1186/s12877-022-03006-6 (PMC9109291; doi:10.1186/s12877-022-03006-6)
Supplement: Supplementary file 1 — Additional file 1. Assessment of methodological limitations. [file 12877_2022_3006_MOESM1_ESM.pdf]

Herrler A, Kukla H, Vennedey V, Stock S. Which features of ambulatory healthcare are preferred by people aged 80 and over? Findings from a systematic review of qualitative studies and appraisal of confidence using GRADE-CERQual. BMC Geriatrics.

Corresponding author: Angélique Herrler, Faculty of Human Sciences and Faculty of Medicine, Graduate School GROW – Gerontological Research on Well-being, University of Cologne, Albertus-Magnus-Platz, 50923 Cologne, Germany; e-mail: angelique.herrler@uni-koeln.de

Additional file 1: Assessment of methodological limitations per finding

**Summary of review finding**	**Studies contributing to the review finding**	**Summary of results**						**Overall Assessment**
		Theoretical Approach	Study Design	Data Collection	Trust-worthiness	Analysis	Ethics	
1. Older people wish to receive care that fits their individual needs.	[1-17]	All adequate	All adequate	All adequate	Most adequate, one unsure	Most adequate, one unsure	All adequate	No or very minor concerns<br><br>The study of King et al. was less trustworthy due to a very one-sided, positive evaluation of the intervention without discussion of the researcher's position. The analysis of Bjornsdottir did not report on discrepant results and was conducted by only one researcher. We had very minor concerns, because these limitations related to only two of seventeen studies and therefore, did not affect the review finding notably.
2. Older people value being looked after regularly.	[2-5, 8, 10, 12-15]	All adequate	All adequate	All adequate	Most adequate, one unsure	Most adequate, one unsure	All adequate	Minor concerns<br><br>The study of King et al. was less trustworthy due to a very one-sided, positive evaluation of the intervention without discussion of the researcher's position. The analysis of Bjornsdottir did not report on discrepant results and was conducted by only one researcher. We had minor concerns, because these limitations related to two of ten studies and weakened the review finding to a limited extent.

Summary of review finding	Studies contributing to the review finding	Summary of results						Overall Assessment
		Theoretical Approach	Study Design	Data Collection	Trust-worthiness	Analysis	Ethics	
3. Older people accept delegation.	[1, 4, 15, 17, 18]<br><br>Abweichend: [1, 15, 18]	All adequate	All adequate	All adequate	All adequate	All adequate	All adequate	No or very minor concerns<br><br>None of the studies contributing to this review finding were found to have notable methodological limitations.
4. Older people value home visits, but not all think they are necessary.	[1, 15, 18]<br><br>Ggf.: [19]	All adequate	All adequate	All adequate	All adequate	All adequate	All adequate	No or very minor concerns<br><br>None of the studies contributing to this review finding were found to have notable methodological limitations.
5. Older people want fast contact to care.	[1, 4, 8, 9, 12, 13, 15, 19]	All adequate	All adequate	All adequate	All adequate	All adequate	All adequate	No or very minor concerns<br><br>None of the studies contributing to this review finding were found to have notable methodological limitations.
6. Older people want easy access to care.	[1, 2, 5, 6, 8, 12, 14, 15, 17, 20]	All adequate	All adequate	All adequate	Most adequate, one unsure	Most adequate, one unsure	All adequate	No or very minor concerns<br><br>The study of King et al. was less trustworthy due to a very one-sided, positive evaluation of the intervention without discussion of the researcher's position. The analysis of Bjornsdottir did not report on discrepant results and was conducted by only one researcher. We had very minor concerns, because these limitations related to two of ten studies, the studies contributed to the review finding only to a small extent and therefore, did not affect the review finding notably.
7. Older people reject waiting times.	[1, 2, 8, 21]	All adequate	All adequate	All adequate	All adequate	Most adequate, one unsure	All adequate	No or very minor concerns<br><br>The analysis of Bjornsdottir did not report on discrepant results and was conducted by only one researcher. We had very minor concerns, because this limitation related to only one of four studies, the study contributed to the review finding only to

Summary of review finding	Studies contributing to the review finding	Summary of results						Overall Assessment
		Theoretical Approach	Study Design	Data Collection	Trust-worthiness	Analysis	Ethics	
								a very small extent and therefore, did not affect the review finding notably.
8. Older people want reliable and continuous care.	[1, 2, 6, 8-12, 14, 15, 17, 18, 21, 22]	All adequate	All adequate	All adequate	All adequate	Most adequate, one unsure	Most adequate, one unsure	No or very minor concerns<br><br>The analysis of Bjornsdottir did not report on discrepant results and was conducted by only one researcher. The study of Martin-Matthews et al. did not report on ethical approval, but the study's aim and results did not seem to be affected by that. We had very minor concerns, because these limitations related to two of fourteen studies, the studies contributed to the review finding only to a small extent and therefore, did not affect the review finding notably.
9. Older people value care coordination.	[4-6, 8, 10, 12-15, 22]	All adequate	All adequate	All adequate	Most adequate, one unsure	All adequate	Most adequate, one unsure	Moderate concerns<br><br>The study of King et al. was less trustworthy due to a very one-sided, positive evaluation of the intervention without discussion of the researcher's position. The study of Martin-Matthews et al. did not report on ethical approval, but the study's aim and results did not seem to be affected by that. We had moderate concerns, because these limitations related to two of ten studies and while the study of Martin-Matthews and Sims-Gould contributed to the review finding only to a very small extent, the limitations in the study of King et al. weakened the confidence in this review finding.
10. Older people prefer home care.	[2, 4, 6, 11, 12, 14, 15, 19, 21]	All adequate	All adequate	All adequate	All adequate	Most adequate, one unsure	All adequate	No or very minor concerns<br><br>The analysis of Bjornsdottir did not report on discrepant results and was only conducted by only one researcher. We had very minor concerns, because this limitation related to only one of nine

Summary of review finding	Studies contributing to the review finding	Summary of results						Overall Assessment
		Theoretical Approach	Study Design	Data Collection	Trust-worthiness	Analysis	Ethics	
								studies, the study contributed to the review finding only to a small extent and therefore, did not affect the review finding notably.
11. Older people prefer personal information.	[1, 8, 15, 17, 19]	All adequate	All adequate	All adequate	All adequate	All adequate	All adequate	No or very minor concerns<br>None of the studies contributing to this review finding were found to have notable methodological limitations.
12. Older people value advice to help with daily life.	[2, 5, 7, 10, 12, 13, 19]	All adequate	All adequate	All adequate	Most adequate, one unsure	Most adequate, one unsure	All adequate	Moderate concerns<br>The analysis of Bjornsdottir did not report discrepant results and was conducted by only one researcher. The study of King et al. was less trustworthy due to a very one-sided, positive evaluation of the intervention without discussion of the researcher's position. We had moderate concerns because these limitations related to two of seven studies and while the study of Bjornsdottir contributed to the review finding only to a very small extent, the limitations in the study of King et al. weakened the confidence in this review finding.
13. Older people want information on care options and services.	[6, 10, 13, 15, 17, 19]	All adequate	All adequate	All adequate	All adequate	All adequate	All adequate	No or very minor concerns<br>None of the studies contributing to this review finding were found to have notable methodological limitations.
14. Older people want to be informed comprehensively.	[1, 2, 6, 8-10, 19, 20]	All adequate	All adequate	All adequate	All adequate	Most adequate, one unsure	All adequate	No or very minor concerns<br>The analysis of Bjornsdottir did not report on discrepant results and was conducted by only one researcher. We had very minor concerns because this limitation related to only one of eight studies, the study contributed to this review finding only to

Summary of review finding	Studies contributing to the review finding	Summary of results						Overall Assessment
		Theoretical Approach	Study Design	Data Collection	Trust-worthiness	Analysis	Ethics	
								a small extent and therefore, did not affect the review finding notably.
15. Older people want more time for their care.	[1, 3-5, 8, 9, 14-18]	All adequate	All adequate	All adequate	Most adequate, one unsure	All adequate	All adequate	No or very minor concerns<br><br>The study of King et al. was less trustworthy due to a very one-sided, positive evaluation of the intervention without discussion of the researcher's position. We had very minor concerns, because this limitation related only to one of eleven studies, the study contributed to this review finding to only a small extent and therefore, did not affect the review finding notably.
16. Older people expect healthcare professionals to be knowledgeable.	[1, 4-6, 8-11, 13, 15, 16, 20]	All adequate	All adequate	All adequate	Most adequate, one unsure	All adequate	All adequate	No or very minor concerns<br><br>The study of King et al. was less trustworthy due to a very one-sided, positive evaluation of the intervention without discussion of the researcher's position. We had very minor concerns, because this limitation related only to one of twelve studies, the study contributed to this review finding to only a small extent and therefore, did not affect the review finding notably.
17. Older people value healthcare professionals' communication skills.	[5, 10-13, 15, 20]	All adequate	All adequate	All adequate	Most adequate, one unsure	All adequate	All adequate	Moderate concerns<br><br>The study of King et al. was less trustworthy due to a very one-sided, positive evaluation of the intervention without discussion of the researcher's position. We had moderate concerns because this limitation related to one of seven studies and therefore, weakened the confidence in this review finding.

Summary of review finding	Studies contributing to the review finding	Summary of results						Overall Assessment
		Theoretical Approach	Study Design	Data Collection	Trust-worthiness	Analysis	Ethics	
18. Older people wish to receive personal attention.	[1-3, 5-7, 9-14, 17, 19, 21, 22]	All adequate	All adequate	All adequate	Most adequate, one unsure	Most adequate, one unsure	Most adequate, one unsure	No or very minor concerns<br><br>The analysis of Bjornsdottir did not report on discrepant results and was conducted by only one researcher. The study of King et al. was less trustworthy due to a very one-sided, positive evaluation of the intervention without discussion of the researcher's position. The study of Martin-Matthews et al. did not report on ethical approval, but the study's aim and results did not seem to be affected by that. We had very minor concerns, because these limitations related to only three of sixteen studies and therefore, did not affect the review finding notably.
19. Older people value close, long-term relationships.	[1, 2, 5-7, 9-11, 14, 18, 21, 22]	All adequate	All adequate	All adequate	Most adequate, one unsure	Most adequate, one unsure	Most adequate, one unsure	Minor concerns<br><br>The analysis of Bjornsdottir did not report on discrepant results and was conducted by only one researcher. The study of King et al. was less trustworthy due to a very one-sided, positive evaluation of the intervention without discussion of the researcher's position. The study of Martin-Matthews et al. did not report on ethical approval, but the study's aim and results did not seem to be affected by that. We had minor concerns, because these limitations related to only three of thirteen studies and therefore, weakened the confidence in the review finding to a limited extent.
20. Older people want to be treated in a friendly way.	[1-3, 5, 7, 9-11, 13, 17, 21]	All adequate	All adequate	All adequate	Most adequate, one unsure	Most adequate, one unsure	All adequate	No or very minor concerns<br><br>The analysis of Bjornsdottir did not report discrepant results and was conducted by only one researcher. The study of King et al. was less trustworthy due to a very one-sided, positive evaluation of the intervention without discussion of

Summary of review finding	Studies contributing to the review finding	Summary of results						Overall Assessment
		Theoretical Approach	Study Design	Data Collection	Trust-worthiness	Analysis	Ethics	
								the researcher's position. We had very minor concerns, because these limitations related to only two of eleven studies, the studies contributed to this review finding only to a small extent and therefore, did not affect the review finding notably.
21. Older people value open and confidential communication.	[1, 5, 6, 8-15, 18, 21]	All adequate	All adequate	All adequate	Most adequate, one unsure	All adequate	All adequate	No or very minor concerns<br>The study of King et al. was less trustworthy due to a very one-sided, positive evaluation of the intervention without discussion of the researcher's position. We had very minor concerns, because this limitation related to only two of thirteen studies, the studies contributed to this review finding only to a very small extent and therefore, did not affect the review finding notably.
22. Older people want to be involved in decisions and care.	[1, 2, 4-6, 8, 9, 11, 12, 14-18, 21, 22]<br>Abweichend: [2, 4, 8, 16]	All adequate	All adequate	All adequate	Most adequate, one unsure	Most adequate, one unsure	Most adequate, one unsure	Minor concerns<br>The analysis of Bjornsdottir did not report on discrepant results and was conducted by only one researcher. The study of King et al. was less trustworthy due to a very one-sided, positive evaluation of the intervention without discussion of the researcher's position. The study of Martin-Matthews et al. did not report on ethical approval, but the study's aim and results did not seem to be affected by that. We had minor concerns, because these limitations related to three of sixteen studies and therefore, weakened the confidence in the review finding to a limited extent.
23. Older people value activity.	[3, 4, 6, 9, 10, 12-15, 19, 20, 22]	All adequate	All adequate	All adequate	All adequate	All adequate	Most adequate, one unsure	No or very minor concerns<br>The study of Martin-Matthews et al. did not report on ethical approval, but the study's aim and results did not seem to be affected by that. We had very

**Summary of review finding**	**Studies contributing to the review finding**	**Summary of results**						**Overall Assessment**
		Theoretical Approach	Study Design	Data Collection	Trust-worthiness	Analysis	Ethics	
								minor concerns because this limitation related to only one of twelve studies, the study contributed to the review finding only to a very small extent and therefore, did not affect the review finding notably.

References

1. Berkelmans PG, Berendsen AJ, Verhaak PF, van der Meer K. Characteristics of general practice care: what do senior citizens value? A qualitative study. BMC Geriatr. 2010;10:80. https://doi.org/10.1186/1471-2318-10-80.

2. Bjornsdottir K. 'Holding on to life': An ethnographic study of living well at home in old age. Nurs Inq. 2018;25(2):1. https://doi.org/10.1111/nin.12228.

3. Faeo SE, Bruvik FK, Tranvag O, Husebo BS. Home-dwelling persons with dementia's perception on care support: Qualitative study. Nurs Ethics. 2020. https://doi.org/10.1177/0969733019893098.

4. Gowing A, Dickinson C, Gorman T, Robinson L, Duncan R. Patients' experiences of a multidisciplinary team-led community case management programme: a qualitative study. BMJ Open. 2016;6(9):e012019. https://doi.org/10.1136/bmjopen-2016-012019.

5. King AII, Boyd ML, Dagley L, Raphael DL. Implementation of a gerontology nurse specialist role in primary health care: Health professional and older adult perspectives. J Clin Nurs. 2018;27(3-4):807-18. https://doi.org/10.1111/jocn.14110.

6. Krothe JS. Constructions of elderly people's perceived needs for community-based long-term care. Indiana University School of Nursing; 1992.

7. Michel T, Helena Lenardt M, Hautsch Willig M, Maria Alvarez A. From real to ideal - the health (un)care of long-lived elders. Rev Bras Enferm. 2015;68(3):343-9. https://doi.org/10.1590/0034-7167.2015680304i.

8. Modig S, Kristensson J, Troein M, Brorsson A, Midlöv P. Frail elderly patients' experiences of information on medication. A qualitative study. BMC Geriatr. 2012;12(1):46. https://doi.org/10.1186/1471-2318-12-46.

9. Moe A, Hellzen O, Enmarker I. The meaning of receiving help from home nursing care. Nurs Ethics. 2013;20(7):737-47. https://doi.org/10.1177/0969733013478959.

10. Sandberg M, Jakobsson U, Midlov P, Kristensson J. Case management for frail older people - a qualitative study of receivers' and providers' experiences of a complex intervention. BMC Health Serv Res. 2014;14. https://doi.org/10.1186/1472-6963-14-14.

11. Soodeen RA, Gregory D, Bond JB. Home care for older couples: "It feels like a security blanket.. ". Qual Health Res. 2007;17(9):1245-55. https://doi.org/10.1177/1049732307307339.

12. Spoorenberg SLW, Wynia K, Fokkens AS, Slotman K, Kremer HPH, Reijneveld SA. Experiences of Community-Living Older Adults Receiving Integrated Care Based on the Chronic Care Model: A Qualitative Study. PLoS One. 2015;10(10):1. https://doi.org/10.1371/journal.pone.0137803.

13. Toien M, Bjork IT, Fagerstrom L. Older users' perspectives on the benefits of preventive home visits. Qual Health Res. 2015;25(5):700-12. https://doi.org/10.1177/1049732314553595.

14. Turjamaa R, Hartikainen S, Kangasniemi M, Pietila AM. Living longer at home: a qualitative study of older clients' and practical nurses' perceptions of home care. J Clin Nurs. 2014;23(21-22):3206-17. https://doi.org/10.1111/jocn.12569.

15. van Blijswijk SCE, de Waard CS, van Peet PG, Keizer D, von Faber M, de Waal MWM, et al. Wishes and needs of community-dwelling older persons concerning general practice: A qualitative study. PLoS One. 2018;13(7):14. https://doi.org/10.1371/journal.pone.0200614.

16. Schulman-Green DJ, Naik AD, Bradley EH, McCorkle R, Bogardus ST. Goal setting as a shared decision making strategy among clinicians and their older patients. Patient Educ Couns. 2006;63(1-2):145-51. https://doi.org/10.1016/j.pec.2005.09.010.

17. Tiilikainen E, Hujala A, Kannasoja S, Rissanen S, Närhi K. "They're always in a hurry" – Older people´s perceptions of access and recognition in health and social care services. Health Soc Care Community. 2019;27(4):1011-8. https://doi.org/10.1111/hsc.12718.

18. van Kempen JA, Robben SH, Zuidema SU, Rikkert MG, Melis RJ, Schers HJ. Home visits for frail older people: a qualitative study on the needs and preferences of frail older people and their informal caregivers. Br J Gen Pract. 2012;62(601):554-60. https://doi.org/10.3399/bjgp12X653606.

19. Behm L, Ivanoff SD, Ziden L. Preventive home visits and health: experiences among very old people. BMC Public Health. 2013;13:378. https://doi.org/10.1186/1471-2458-13-378.

20. Walker R, Ratcliffe J, White A, Visvanathan R. Dementia assessment services: What are the perceptions of older people? Australas J Ageing. 2018;37(1):43-7. https://doi.org/10.1111/ajag.12455.

21. Jarling A, Rydstrom I, Ernsth-Bravell M, Nystrom M, Dalheim-Englund AC. Becoming a guest in your own home: Home care in Sweden from the perspective of older people with multimorbidities. Int J Older People Nurs. 2018;13(3). https://doi.org/10.1111/opn.12194.

22. Martin-Matthews A, Sims-Gould J. Employers, home support workers and elderly clients: identifying key issues in delivery and receipt of home support. Healthc Q. 2008;11(4):69-75. https://doi.org/10.12927/hcq.2008.20073.
