# Supplementary material for: Which features of ambulatory healthcare are preferred by people aged 80 and over? Findings from a systematic review of qualitative studies and appraisal of confidence using GRADE-CERQual
Source: BMC Geriatr. 2022 May 16;22:428. doi: 10.1186/s12877-022-03006-6 (PMC9109291; doi:10.1186/s12877-022-03006-6)
Supplement: Supplementary file 2 — Additional file 2. Assessment of coherence and adequacy. [file 12877_2022_3006_MOESM2_ESM.pdf]

Herrler A, Kukla H, Vennedey V, Stock S. Which features of ambulatory healthcare are preferred by people aged 80 and over? Findings from a systematic review of qualitative studies and appraisal of confidence using GRADE-CERQual. BMC Geriatrics.

Corresponding author: Angélique Herrler, Faculty of Human Sciences and Faculty of Medicine, Graduate School GROW – Gerontological Research on Well-being, University of Cologne, Albertus-Magnus-Platz, 50923 Cologne, Germany; e-mail: angelique.herrler@uni-koeln.de

Additional file 2: Assessment of coherence and adequacy

For these domains, the review findings were also compared with contributing material from the respective studies which was available via MAXQDA. The MAXQDA data is available from the authors on request.

Review finding	Studies contributing to the review finding	Concerns about coherence	Concerns about adequacy
**1. Older people wish to receive care that fits their individual needs.**<br>For older people, one remarkable feature was that they wanted to receive holistic care, sufficient support that met their needs and supported their independence, and individual adjustments to care and care planning [1-15]. Concomitantly, older people negatively judged experiences such as not receiving the care needed (regarding lack of time, money, personnel or individual attention), not receiving individual adjustments to care, not been taken seriously with their concerns, and a focus on acute problems and symptoms instead of a long-term perspective and goal setting [2, 4, 6-9, 11, 15-17].	[1-17]	**Minor concerns**<br>While approximately two thirds of underlying data directly supported this review finding, one third rather indirectly described the core of the finding or did not explore it in detail. However, there were no contradictory data.	**No or very minor concerns**<br>Seventeen studies contributed to this review finding and represented a variety of examples that illustrated or explained the finding. Although some findings did not directly describe the review finding (see coherence), the other findings still provided rich underlying data.
**2. Older people value being looked after regularly**.<br>The older people highly appreciated having a healthcare professional who “kept an eye” on them. They felt reassured when someone monitored their health status, looked after them regularly, cared for chronic issues (e.g., wounds, medications) and could intervene fast, if	[2-5, 8, 10, 12-15]	**Minor concerns**<br>While approximately two thirds of underlying data directly supported this review finding, one third rather indirectly described the core of the	**No or very minor concerns**<br>Ten studies contributed to this review finding and represented a variety of examples that illustrate or explained the finding. Although

Review finding	Studies contributing to the review finding	Concerns about coherence	Concerns about adequacy
necessary [2-5, 8, 10, 12-15]. However, a specific frequency or contact interval was not proposed.		finding or did not explore it in detail. However, there were no contradictory data.	some findings did not directly describe the review finding, the other findings still provided rich underlying data.
**3. Older people accept delegation.**<br>Regarding general care practices and home visits, most older people accepted task delegation to assistants or nurses, or even welcomed it. On the condition that this person provided a continuous contact, knew them well and exchanged information with a GP or specialist, delegation was found to be a good alternative for minor problems or follow-up appointments, and could even mean that more time and attention was provided for the older person [1, 4, 15, 17, 18]. Nevertheless, some older people preferred contact with a physician and sometimes considered nurses and assistants to be barriers to physician access [1, 15, 18].	[1, 4, 15, 17, 18]	**Moderate concerns**<br>Most underlying data directly referred to this review finding and the studies in question reported that the majority of their participants accepted delegation. However, this was subject to certain conditions and three out of five studies also reported deviating opinions of their participants.	**Moderate concerns**<br>Only five studies contributed to this review finding and some of them reported different views regarding delegation. Although studies described that "some" or "most" of their participants represented the according views, this was not clearly quantifiable. However, the underlying data delivered sufficient explanation to the core of this this review finding.
**4. Older people value home visits, but not all think they are necessary.**<br>Home visits were discussed controversially in the qualitative studies. In general, it became apparent that receiving a home visit was seen as favorable if someone really needed it but was not required in less urgent cases [1, 18]. Nevertheless, home visits were welcomed as offering the potential for personal attention and as providing more information on the older person's living circumstances and psychosocial context [1, 15, 18]. By contrast, one study on preventive home visits found that these could be too demanding for some ill people [19].	[1, 15, 18, 19]	**Moderate concerns**<br>Most underlying data directly referred to this review finding. However, it was not clear in which cases home visits were only a useful "add-on" to their healthcare and in which they were really necessary. We described this as moderate instead of serious concern, because the conditions and variety of opinions in general were part of the review finding's	**Moderate concerns**<br>Only four studies contributed to this review findings. Although the underlying data sufficiently explained the different motives in the valuation of home visits, the primary studies' did not quantify the participants representing the according views.

Review finding	Studies contributing to the review finding	Concerns about coherence	Concerns about adequacy
		description and already referred to the ambiguous data.	
**5. Older people want fast contact to care.**<br><br>It became apparent that older people wish to make contact with a (known) healthcare professional fast, if needed. They prefer to have a constant person or healthcare practice which they could contact if advice or help was required [1, 9, 12, 13, 19]. Widespread and fast availability via phone was especially valued [1, 4, 8, 9, 13, 15].	[1, 4, 8, 9, 12, 13, 15, 19]	**No or very minor concerns**<br><br>The vast majority of underlying data directly and unambiguously supported the review finding and there were no contradictory data.	**No or very minor concerns**<br><br>Eight studies contributed to this review finding and most of their findings also included rich explanations about why older people wanted fast contact to care.
**6. Older people want easy access to care**.<br><br>Most participants in the qualitative studies had already experienced difficulties or restrictions in accessing healthcare, e.g., for specialist services. Although some of them mentioned that the proximity of services was good, widespread access to healthcare, including on weekends and on an intermittent basis, as well as easily accessible follow-up services and referrals, were rated most important [1, 2, 6, 8, 12, 15, 17, 20]. Older people identified restricted opening hours, the fragmented nature of the care systems, and the need to go through several levels of care before receiving the right treatment as barriers to good access [6, 12, 14, 15, 17, 20].	[1, 2, 5, 6, 8, 12, 14, 15, 17, 20]	**No or very minor concerns**<br><br>The vast majority of underlying data directly and unambiguously supported the review finding and there were no contradictory data. The meaning of "access" differed between the primary studies sometimes, but this was considered in the review finding's description.	**No or very minor concerns**<br><br>Ten studies contributed to this review finding and most of their findings included an explanation what easy access meant to the participants.
**7. Older people reject waiting times.**<br><br>The older participants found waiting times (waiting for telephone contact, waiting for an appointment, waiting at an appointment) generally problematic [1, 2, 7, 8, 21]. Some explained that their issues were urgent and priority should be given to old age; inconvenience, such as hard benches in waiting rooms, was also mentioned in connection with waiting times [1, 7].	[1, 2, 8, 21]	**No or very minor concerns**<br><br>The underlying data directly and unambiguously supported the review finding and there were no contradictory data.	**Minor concerns**<br><br>Only four studies contributed to this review finding, but these studies provided a variety of examples and descriptions on the negative perception of waiting times.

Review finding	Studies contributing to the review finding	Concerns about coherence	Concerns about adequacy
**8. Older people want reliable and continuous care**.<br><br>Older people frequently reported a high turnover of healthcare professionals responsible for them but strongly desired continuity. They wanted their caregivers to know them personally and to have a good overview of their living circumstances and care needs [1, 4, 6, 8-12, 14, 15, 17, 18, 21, 22]. A lack of continuity led to stress, unstructured and impersonal care, insecurity and information loss [6, 8, 9, 14, 21, 22]. Furthermore, they wanted reliability in receiving care, e.g., a regular schedule and predictability [2, 13, 21, 22].	[1, 2, 6, 8-12, 14, 15, 17, 18, 21, 22]	**No or very minor concerns**<br><br>The vast majority of underlying data directly and unambiguously supported the review finding and there were no contradictory data.	**No or very minor concerns**<br><br>Fourteen studies contributed to this review finding and represented a variety of examples that illustrated or explained the finding.
**9. Older people value care coordination**.<br><br>Older people greatly appreciated care coordination, including in the form of case management. They felt reassured when their care services, treatments, collaboration between different providers and necessary adaptations were organized and managed by a healthcare professional, someone who had an overview and was able to provide them with additional support, where needed [4-6, 8, 10, 12-15, 22].	[4-6, 8, 10, 12-15, 22]	**No or very minor concerns**<br><br>Although only half of the underlying data directly supported the review finding, the other half did so by describing the wish for a person that helped them manage their issues and there were no contradictory data.	**No or very minor concerns**<br><br>Ten studies contributed to this review finding and represented a variety of examples that illustrated or explained the finding.
**10. Older people prefer home care.**<br><br>Consistently, study participants expressed a strong desire to stay in their own homes for as long as possible because of the better quality of life, increased privacy and control, and the belief that their homes offered a more secure environment [2, 4, 6, 11, 12, 15, 19, 21]. They acknowledged that receiving home care and support was needed for them to age in place [2, 6, 11, 12, 14]. There were indications that, in contrast, a nursing home would constitute a threat to older people's personal integrity and quality of life; they had quite a negative view of	[2, 4, 6, 11, 12, 14, 15, 19, 21]	**Minor concerns**<br><br>The vast majority of underlying data directly and unambiguously supported the review finding. However, in the study of Gowing et al., it was described that some participants preferred the hospital under certain conditions.	**No or very minor concerns**<br><br>Nine studies contributed to this review finding and represented a variety of examples that illustrated or explained the finding.

Review finding	Studies contributing to the review finding	Concerns about coherence	Concerns about adequacy
institutional care [6, 12]. Seldom was institutional care perceived as the better option to meet their needs [4].			
**11. Older people prefer personal information.**<br><br>Older people found it easier to understand information in a face-to-face-conversation, where questions and difficult terms or issues can be discussed directly; brochures or leaflets were requested rather as memory aids [1, 8, 19]. According to the results of two studies, offers of digital services or online communication were refused [15, 17].	[1, 8, 15, 17, 19]	**Minor concerns**<br><br>Although the majority of underlying data directly supported the review finding, two studies suggested that written information can be an important, too. However, this was described as a supplementary to support personally communicated information.	**Minor concerns**<br><br>Minor concerns. Only five studies contributed to this review finding but they provided a variety of examples and descriptions on the advantage of personal information.
**12. Older people value advice to help with daily life**.<br><br>Several studies found that older people value receiving advice to help with their daily lives. They welcomed practical advice for adaptations of their home (e.g., the removal of carpets to prevent falls), safety information and education regarding health issue prevention and diet, and recommendations for exercises [2, 5, 7, 10, 12, 13, 19].	[2, 5, 7, 10, 12, 13, 19]	**No or very minor concerns**<br><br>The vast majority of underlying data directly and unambiguously supported the review finding and there were no contradictory data.	**No or very minor concerns**<br><br>Seven studies contributed to this review finding and represented a variety of examples that illustrated or explained the finding.
**13. Older people want information on care options and services.**<br><br>The studies' participants wanted to receive information on care options, services and additional help, in terms of which were suitable and available for them, where they could be accessed and how they could apply for them [6, 10, 13, 15, 17, 19].	[6, 10, 13, 15, 17, 19]	**Minor concerns**<br><br>While approximately the half of underlying data directly supported this review finding, the other half did so rather indirectly. However, there were no contradictory data and the indirectly supporting half of	**Minor concerns**<br><br>Only six studies contributed to this review finding but the primary studies provided a variety of examples and descriptions on the usefulness and desirability of information on care options and services.

Review finding	Studies contributing to the review finding	Concerns about coherence	Concerns about adequacy
		data also illustrated examples in favor of the review finding.	
**14. Older people want to be informed comprehensively.** Older people wished to be informed well about their health status, treatments and further issues by healthcare professionals so that they can understand the procedures [1, 2, 6, 8-10, 19, 20]. In contrast, it was reported that some did not wish for more explanations and that they were satisfied with limited information [1, 8].	[1, 2, 6, 8-10, 19, 20]	**Moderate concerns** Only half of the underlying data directly referred to the wish of being informed comprehensively. Moreover, within some of the studies, it was described that some older people were satisfied with limited information or scared by too much information. However, we described this as moderate instead of serious concern, because the conditions and variety of opinions in general were part of the review finding's description and already referred to the ambiguous data.	**Moderate concerns** Eight studies contributed to this review finding and represented a variety of examples and descriptions about the wish to receive comprehensive information. However, the study of Modig et al. contributed the largest part of data to this finding and the other contributing studies did not provide the same level of data richness as Modig et al.
**15. Older people want more time for their care.** A major obstacle to favorable care was time constraints. Participants described that their care or medical appointments were frequently rushed and that there was not enough time for the necessary help and conversation [1, 3-5, 8, 9, 14-18]. Insufficient time for care was described as resulting in unresolved questions and a focus on acute tasks and symptoms, rather than on considering long-term plans and goals [3, 4, 8, 16, 17].	[1, 3-5, 8, 9, 14-18]	**No or very minor concerns** The majority of underlying data directly and unambiguously supported the review finding and there were no contradictory data.	**No or very minor concerns** Eleven studies contributed to this review finding and represented a variety of examples that illustrated or explained the finding.
**16. Older people expect healthcare professionals to be knowledgeable**.	[1, 4-6, 8-11, 13, 15, 16, 20]	**No or very minor concerns** The majority of underlying data directly and unambiguously	**No or very minor concerns** Twelve studies contributed to this review finding and represented a

Review finding	Studies contributing to the review finding	Concerns about coherence	Concerns about adequacy
The older people expected healthcare professionals to have a certain level of knowledge and experience in order to provide good care, which was also described as a condition for trust [1, 4-6, 8-11, 13, 15, 16, 20].		supported the review finding and there were no contradictory data.	variety of examples that illustrated or explained the finding.
**17. Older people value healthcare professionals' communication skills.**<br>Older people valued interpersonal and educational skills, e.g., regarding explanations of treatment. Healthcare professionals that were "good communicators" helped improve the understanding of care and affected older people positively, e.g., by lessening anxiety [5, 10-13, 15, 20].	[5, 10-13, 15, 20]	**No or very minor concerns**<br>The majority of underlying data directly and unambiguously supported the review finding and there were no contradictory data.	**Minor concerns**<br>Seven studies contributed to this review finding and represented a variety of examples and descriptions that supported the review finding. However, half of the underlying data were provided by only one study (King et al.).
**18. Older people wish to receive personal attention.**<br>Descriptions of care as an important social contact point were relevant in almost all studies, but more frequently in those describing home care and home visits. The interviewees appreciated having the feeling that someone was interested and cared about them [1, 5-7, 12, 19]. In several studies, it was indicated that the social aspects of care – caregivers spending time with them, starting conversations, providing emotional support – were highly valuable for the older persons' well-being [3, 5, 6, 9-14, 17, 19, 21, 22]. The older people also revealed this wish for beneficial contacts in describing negative experiences, e.g., caregivers visibly hurrying, not talking and not focusing on them, which resulted in negative feelings and a sense of isolation [2, 6, 9, 11, 12, 17].	[1-3, 5-7, 9-14, 17, 19, 21, 22]	**No or very minor concerns**<br>The vast majority of underlying data directly and unambiguously supported the review finding and there were no contradictory data.	**No or very minor concerns**<br>Sixteen studies contributed to this review finding and represented a variety of examples that illustrated or explained the finding.

Review finding	Studies contributing to the review finding	Concerns about coherence	Concerns about adequacy
**19. Older people value close, long-term relationships.**<br><br>Establishing close, long-term care relationships was an overall present topic, although mainly related to home care professionals (e.g., nurses) or case managers. Older people wanted trustful interactions with well-known healthcare professionals that enabled them to share personal issues and to feel safe and strengthened [1, 2, 5-7, 9-11, 14, 18, 21]. Frequently, it was indicated that they developed friendships or family-like relationships [2, 5, 9-11, 21, 22].	[1, 2, 5-7, 9-11, 14, 18, 21, 22]	**No or very minor concerns**<br><br>The vast majority of underlying data directly and unambiguously supported the review finding and there were no contradictory data.	**No or very minor concerns**<br><br>Twelve studies contributed to this review finding and represented a variety of examples that illustrated or explained the finding.
**20. Older people want to be treated in a friendly way.**<br><br>Older people valued a kind, open and positive attitude on the part of caregivers and wanted to be treated respectfully [1, 3, 5, 7, 9-11, 13, 17]. On the other hand, some studies described how older people felt hurt when caregivers were authoritative, disrespectful, impersonal, rude or – in general – lacked empathy [2, 9, 17, 21].	[1-3, 5, 7, 9-11, 13, 17, 21]	**No or very minor concerns**<br><br>The vast majority of underlying data directly and unambiguously supported the review finding and there were no contradictory data.	**No or very minor concerns**<br><br>Eleven studies contributed to this review finding and represented a variety of examples that illustrated or explained the finding.
**21. Older people value open and confidential communication.**<br><br>Older people would like to communicate with their care providers in an open and confidential manner. The importance of trust, genuine interest and attention to the person's broader health concerns and living circumstances were stressed, as well as the possibility of discussing everything with the professionals [1, 5, 10-15, 18, 21]. Concomitantly, the studies' participants described negative experiences, such as professionals not listening to them, not having the chance to speak about personal problems, and feelings of distrust, shame or being a burden, which resulted in inhibited communication [6, 8, 9, 15, 21].	[1, 5, 6, 8-15, 18, 21]	**No or very minor concerns**<br><br>The majority of underlying data directly and unambiguously supported the review finding and there were no contradictory data.	**No or very minor concerns**<br><br>Thirteen studies contributed to this review finding and represented a variety of examples that illustrated or explained the finding.

Review finding	Studies contributing to the review finding	Concerns about coherence	Concerns about adequacy
**22. Older people want to be involved in decisions and care.**<br><br>The majority of studies indicated that older persons wanted to be involved in decision-making and planning regarding their healthcare and lifestyle as autonomous and equal partners [2, 4-6, 8, 9, 11, 12, 14-18, 21, 22]. This was described as a wish to be asked about needs and priorities, instead of professionals assuming that they knew what these were, and as a wish to be taken seriously [1, 6, 12, 15, 16, 21]. On the other hand, professionals not taking older people's perspective into account, acting in a paternalistic way and not discussing individual concerns or goals were judged negatively [5, 9, 16, 17]. Nevertheless, the minority of the older people wanted to be rather passive, relied on care professionals and wanted them to provide care and make decisions, e.g., regarding hospital admission [2, 4, 8, 16].	[1, 2, 4-6, 8, 9, 11, 12, 14-18, 21, 22]	**Minor concerns**<br><br>The majority of underlying data directly referred to the wish of being involved in decisions and care, but within some of the studies, it was described that some older people wished to take a passive role. However, we described this as minor instead of moderate concern, because the conditions and variety of opinions in general were part of the review finding's description, and the ambiguous data clearly represented a minority.	**No or very minor concerns**<br><br>Sixteen studies contributed to this review finding and represented a variety of examples that illustrated or explained the finding.
**23. Older people value activity.**<br><br>Several participants expressed the wish to remain as active as possible, e.g., regarding physical activity, volunteer work or social activities. They appreciated care professionals who supported them doing so [6, 9, 10, 12, 13, 15]. Furthermore, the older persons found it highly valuable when care professionals motivated them to improve their health and living circumstances, opened up a new, positive perspective of their possibilities and encouraged them to take on active roles [3, 4, 10, 12-14, 19, 20, 22].	[3, 4, 6, 9, 10, 12-15, 19, 20, 22]	**No or very minor concerns**<br><br>The majority of underlying data directly and unambiguously supported the review finding and there were no contradictory data.	**No or very minor concerns**<br><br>Twelve studies contributed to this review finding and represented a variety of examples that illustrated or explained the finding.

References

1. Berkelmans PG, Berendsen AJ, Verhaak PF, van der Meer K. Characteristics of general practice care: what do senior citizens value? A qualitative study. BMC Geriatr. 2010;10:80. https://doi.org/10.1186/1471-2318-10-80.

2. Bjornsdottir K. 'Holding on to life': An ethnographic study of living well at home in old age. Nurs Inq. 2018;25(2):1. https://doi.org/10.1111/nin.12228.

3. Faeo SE, Bruvik FK, Tranvag O, Husebo BS. Home-dwelling persons with dementia's perception on care support: Qualitative study. Nurs Ethics. 2020. https://doi.org/10.1177/0969733019893098.

4. Gowing A, Dickinson C, Gorman T, Robinson L, Duncan R. Patients' experiences of a multidisciplinary team-led community case management programme: a qualitative study. BMJ Open. 2016;6(9):e012019. https://doi.org/10.1136/bmjopen-2016-012019.

5. King AII, Boyd ML, Dagley L, Raphael DL. Implementation of a gerontology nurse specialist role in primary health care: Health professional and older adult perspectives. J Clin Nurs. 2018;27(3-4):807-18. https://doi.org/10.1111/jocn.14110.

6. Krothe JS. Constructions of elderly people's perceived needs for community-based long-term care. Indiana University School of Nursing; 1992.

7. Michel T, Helena Lenardt M, Hautsch Willig M, Maria Alvarez A. From real to ideal - the health (un)care of long-lived elders. Rev Bras Enferm. 2015;68(3):343-9. https://doi.org/10.1590/0034-7167.2015680304i.

8. Modig S, Kristensson J, Troein M, Brorsson A, Midlöv P. Frail elderly patients' experiences of information on medication. A qualitative study. BMC Geriatr. 2012;12(1):46. https://doi.org/10.1186/1471-2318-12-46.

9. Moe A, Hellzen O, Enmarker I. The meaning of receiving help from home nursing care. Nurs Ethics. 2013;20(7):737-47. https://doi.org/10.1177/0969733013478959.

10. Sandberg M, Jakobsson U, Midlov P, Kristensson J. Case management for frail older people - a qualitative study of receivers' and providers' experiences of a complex intervention. BMC Health Serv Res. 2014;14. https://doi.org/10.1186/1472-6963-14-14.

11. Soodeen RA, Gregory D, Bond JB. Home care for older couples: "It feels like a security blanket.. ". Qual Health Res. 2007;17(9):1245-55. https://doi.org/10.1177/1049732307307339.

12. Spoorenberg SLW, Wynia K, Fokkens AS, Slotman K, Kremer HPH, Reijneveld SA. Experiences of Community-Living Older Adults Receiving Integrated Care Based on the Chronic Care Model: A Qualitative Study. PLoS One. 2015;10(10):1. https://doi.org/10.1371/journal.pone.0137803.

13. Toien M, Bjork IT, Fagerstrom L. Older users' perspectives on the benefits of preventive home visits. Qual Health Res. 2015;25(5):700-12. https://doi.org/10.1177/1049732314553595.

14. Turjamaa R, Hartikainen S, Kangasniemi M, Pietila AM. Living longer at home: a qualitative study of older clients' and practical nurses' perceptions of home care. J Clin Nurs. 2014;23(21-22):3206-17. https://doi.org/10.1111/jocn.12569.

15. van Blijswijk SCE, de Waard CS, van Peet PG, Keizer D, von Faber M, de Waal MWM, et al. Wishes and needs of community-dwelling older persons concerning general practice: A qualitative study. PLoS One. 2018;13(7):14. https://doi.org/10.1371/journal.pone.0200614.

16. Schulman-Green DJ, Naik AD, Bradley EH, McCorkle R, Bogardus ST. Goal setting as a shared decision making strategy among clinicians and their older patients. Patient Educ Couns. 2006;63(1-2):145-51. https://doi.org/10.1016/j.pec.2005.09.010.

17. Tiilikainen E, Hujala A, Kannasoja S, Rissanen S, Närhi K. "They're always in a hurry" – Older people´s perceptions of access and recognition in health and social care services. Health Soc Care Community. 2019;27(4):1011-8. https://doi.org/10.1111/hsc.12718.

18. van Kempen JA, Robben SH, Zuidema SU, Rikkert MG, Melis RJ, Schers HJ. Home visits for frail older people: a qualitative study on the needs and preferences of frail older people and their informal caregivers. Br J Gen Pract. 2012;62(601):554-60. https://doi.org/10.3399/bjgp12X653606.

19. Behm L, Ivanoff SD, Ziden L. Preventive home visits and health: experiences among very old people. BMC Public Health. 2013;13:378. https://doi.org/10.1186/1471-2458-13-378.

20. Walker R, Ratcliffe J, White A, Visvanathan R. Dementia assessment services: What are the perceptions of older people? Australas J Ageing. 2018;37(1):43-7. https://doi.org/10.1111/ajag.12455.

21. Jarling A, Rydstrom I, Ernsth-Bravell M, Nystrom M, Dalheim-Englund AC. Becoming a guest in your own home: Home care in Sweden from the perspective of older people with multimorbidities. Int J Older People Nurs. 2018;13(3). https://doi.org/10.1111/opn.12194.

22. Martin-Matthews A, Sims-Gould J. Employers, home support workers and elderly clients: identifying key issues in delivery and receipt of home support. Healthc Q. 2008;11(4):69-75. https://doi.org/10.12927/hcq.2008.20073.
