# Supplementary material for: Which features of ambulatory healthcare are preferred by people aged 80 and over? Findings from a systematic review of qualitative studies and appraisal of confidence using GRADE-CERQual
Source: BMC Geriatr. 2022 May 16;22:428. doi: 10.1186/s12877-022-03006-6 (PMC9109291; doi:10.1186/s12877-022-03006-6)
Supplement: Supplementary file 3 — Additional file 3. Assessment of relevance. [file 12877_2022_3006_MOESM3_ESM.pdf]

Herrler A, Kukla H, Vennedey V, Stock S. Which features of ambulatory healthcare are preferred by people aged 80 and over? Findings from a systematic review of qualitative studies and appraisal of confidence using GRADE-CERQual. BMC Geriatrics.

Corresponding author: Angélique Herrler, Faculty of Human Sciences and Faculty of Medicine, Graduate School GROW – Gerontological Research on Well-being, University of Cologne, Albertus-Magnus-Platz, 50923 Cologne, Germany; e-mail: angelique.herrler@uni-koeln.de

Additional file 3: Assessment of relevance per finding

1. Older people wish to receive care that fits their individual needs. [1-17]

**Dimensions of context**	**Assessment of relevance**			
	**Direct relevance**	**Indirect relevance**	**Partial relevance**	**Uncertain relevance**
**Phenomenon of interest**: Preferences and wishes regarding favorable aspects of ambulatory healthcare	[1, 3, 6, 15, 17]	[2, 4, 5, 7-14, 16]<br><br>Studies focus on experiences and perceptions, but preferences and wishes are/can be derived from that		
**Population/perspective**: people aged 80 and over	[1-17]			
**Care setting**: ambulatory healthcare, outpatient healthcare, home healthcare	[1-7, 9-15]		[8, 17]<br><br>In both studies, parts of data could possibly not have been clearly differentiated from inpatient/institutional care and social services	[16]<br><br>Parts of data could possibly not have been clearly differentiated from inpatient/institutional care
**Type of care**: professional medical and nursing care	[1-5, 7-10, 12-17]		[6, 11]<br><br>In both studies, parts of data could possibly not have been clearly differentiated from services not provided by healthcare professionals, such as housekeeping	
**CERQual assessment of relevance**	**Minor concerns**. Although the phenomenon of interest in the majority of studies was of indirect relevance (experiences and descriptions instead of preferences and wishes), preferences and wishes were or could be derived from them directly and therefore, we did not have concerns that this negatively impacted the confidence in the review finding. However, in five out of sixteen studies, parts of data could possibly not have been clearly differentiated from services not provided by healthcare professionals or in institutional settings. Since these studies only represented a small part of contributing data, our concerns regarding the impact on relevance of this review finding remained minor.			

**2. Older people value being looked after regularly. [2-5, 8, 10, 12-15]**				
**Dimensions of context**	**Assessment of relevance**			
	**Direct relevance**	**Indirect relevance**	**Partial relevance**	**Uncertain relevance**
**Phenomenon of interest**: Preferences and wishes regarding favorable aspects of ambulatory healthcare	[3, 15]	[2, 4, 5, 8, 10, 12-14]		
**Population/perspective**: people aged 80 and over	[2-5, 8, 10, 12-15]			
**Care setting**: ambulatory healthcare, outpatient healthcare, home healthcare	[2-5, 10, 12-15]		[8]<br><br>Parts of data could possibly not have been clearly differentiated from inpatient/institutional care	
**Type of care**: professional medical and nursing care	[2-5, 8, 10, 12-15]			
**CERQual assessment of relevance**	**No or very minor concerns.** Although the phenomenon of interest in the majority of studies was of indirect relevance (experiences and descriptions instead of preferences and wishes), preferences and wishes were or could be derived from them directly. The fact that parts of data could possibly not have been clearly differentiated from services not provided by healthcare professionals or in institutional settings was only subject to one out of ten studies. Moreover, this study contributed only a very small part of data to the review finding. Therefore, we did not have concerns that there were negative impacts on the confidence in this review finding.			

**3. Older people accept delegation. [1, 4, 15, 17, 18]**				
**Dimensions of context**	**Assessment of relevance**			
	**Direct relevance**	**Indirect relevance**	**Partial relevance**	**Uncertain relevance**
**Phenomenon of interest**: Preferences and wishes regarding favorable aspects of ambulatory healthcare	[1, 15, 17, 18]	[4]		
**Population/perspective**: people aged 80 and over	[1, 4, 15, 17, 18]			
**Care setting**: ambulatory healthcare, outpatient healthcare, home healthcare	[1, 4, 15, 18]		[17]<br><br>Parts of data could possibly not have been clearly differentiated from inpatient/institutional care and social services	
**Type of care**: professional medical and nursing care	[1, 4, 15, 17, 18]			
**CERQual assessment of relevance**	**Minor concerns.** Although the phenomenon of interest studied in Gowing et al. was of indirect relevance (experiences and descriptions instead of preferences and wishes), preferences and wishes were or could be derived from them directly. However, in another study, parts of data could possibly not have been clearly differentiated from services not provided by healthcare professionals or in institutional settings. Although this study contributed only a small part of data to this review finding, two out of five studies were affected by indirect or partial relevance, so we had minor concerns that this negatively impacted the confidence in this review finding.			

**4. Older people value home visits, but not all think they are necessary. [1, 15, 18, 19]**				
**Dimensions of context**	**Assessment of relevance**			
	**Direct relevance**	**Indirect relevance**	**Partial relevance**	**Uncertain relevance**
**Phenomenon of interest**: Preferences and wishes regarding favorable aspects of ambulatory healthcare	[1, 15, 18]	[19]		
**Population/perspective**: people aged 80 and over	[1, 15, 18, 19]			
**Care setting**: ambulatory healthcare, outpatient healthcare, home healthcare	[1, 15, 18, 19]			
**Type of care**: professional medical and nursing care	[1, 15, 18, 19]			
**CERQual assessment of relevance**	**No or very minor concerns.** Although the phenomenon of interest in one out of four studies was of indirect relevance (experiences and descriptions instead of preferences and wishes), preferences and wishes were or could be derived directly and therefore, we did not have concerns that this negatively impacted the confidence in the review finding.			

**5. Older people want fast contact to care. [1, 4, 8, 9, 12, 13, 15, 19]**				
**Dimensions of context**	**Assessment of relevance**			
	**Direct relevance**	**Indirect relevance**	**Partial relevance**	**Uncertain relevance**
**Phenomenon of interest**: Preferences and wishes regarding favorable aspects of ambulatory healthcare	[1, 15]	[4, 8, 9, 12, 13, 19]		
**Population/perspective**: people aged 80 and over	[1, 4, 8, 9, 12, 13, 15, 19]			
**Care setting**: ambulatory healthcare, outpatient healthcare, home healthcare	[1, 4, 9, 12, 13, 15, 19]		[8]<br><br>Parts of data could possibly not have been clearly differentiated from inpatient/institutional care	
**Type of care**: professional medical and nursing care	[1, 4, 8, 9, 12, 13, 15, 19]			
**CERQual assessment of relevance**	**No or very minor concerns.** Although the phenomenon of interest in the majority of studies was of indirect relevance (experiences and descriptions instead of preferences and wishes), preferences and wishes were or could be derived from them directly and therefore, we did not have concerns that this negatively impacted the confidence in the review finding. In the study of Modig et al., parts of data could possibly not have been clearly differentiated from services not provided by healthcare professionals or in institutional settings. However, this was subject to only one out of eight studies and the contribution of data from this study was very small.			

6. **Older people want easy access to care. [1, 2, 5, 6, 8, 12, 14, 15, 17, 20]**				
**Dimensions of context**	**Assessment of relevance**			
	**Direct relevance**	**Indirect relevance**	**Partial relevance**	**Uncertain relevance**
**Phenomenon of interest**: Preferences and wishes regarding favorable aspects of ambulatory healthcare	[1, 6, 15, 17, 20]	[2, 5, 8, 12, 14]		
**Population/perspective**: people aged 80 and over	[1, 2, 5, 6, 8, 12, 14, 15, 17, 20]			
**Care setting**: ambulatory healthcare, outpatient healthcare, home healthcare	[1, 2, 5, 6, 12, 14, 15]		[8, 17, 20]<br><br>In all three studies, parts of data could possibly not have been clearly differentiated from inpatient/institutional care and social services	
**Type of care**: professional medical and nursing care	[1, 2, 5, 8, 12, 14, 15, 17, 20]		[6]<br><br>Parts of data could possibly not have been clearly differentiated from services not provided by healthcare professionals, such as housekeeping	
**CERQual assessment of relevance**	**Moderate concerns.** Although the phenomenon of interest in approximately half of the studies was of indirect relevance (experiences and descriptions instead of preferences and wishes), preferences and wishes were or could be derived from them directly and therefore, we did not have concerns that this negatively impacted the confidence in the review finding. However, in four out of ten studies, parts of data could possibly not have been clearly differentiated from institutional care or services not provided by healthcare professionals. Since two of them (Tiilikainen et al. and Walker et al.) contributed a notable part of data to this review finding, we had moderate concerns about the impact on confidence.			

7. **Older people reject waiting times. [1, 2, 8, 21]**				
**Dimensions of context**	**Assessment of relevance**			
	**Direct relevance**	**Indirect relevance**	**Partial relevance**	**Uncertain relevance**
**Phenomenon of interest**: Preferences and wishes regarding favorable aspects of ambulatory healthcare	[1]	[2, 8, 21]		
**Population/perspective**: people aged 80 and over	[1, 2, 8, 21]			
**Care setting**: ambulatory healthcare, outpatient healthcare, home healthcare	[1, 2, 21]		[8]<br><br>Parts of data could possibly not have been clearly differentiated from inpatient/institutional care	
**Type of care**: professional medical and nursing care	[1, 2, 8, 21]			
**CERQual assessment of relevance**	**Minor concerns.** Although the phenomenon of interest in the majority of studies was of indirect relevance (experiences and descriptions instead of preferences and wishes), preferences and wishes were or could be derived from them directly and therefore, we did not have concerns that this negatively impacted the confidence in the review finding. However, in the study of Modig et al., parts of data could possibly not have been clearly differentiated from services not provided by healthcare professionals or in institutional settings and this may have affected the strength of the review finding.			

**8. Older people want reliable and continuous care. [1, 2, 6, 8-12, 14, 15, 17, 18, 21, 22]**				
**Dimensions of context**	**Assessment of relevance**			
	**Direct relevance**	**Indirect relevance**	**Partial relevance**	**Uncertain relevance**
**Phenomenon of interest**: Preferences and wishes regarding favorable aspects of ambulatory healthcare	[1, 6, 15, 17, 18]	[2, 8-12, 14, 21, 22]		
**Population/perspective**: people aged 80 and over	[1, 2, 6, 8-12, 14, 15, 17, 18, 21, 22]			
**Care setting**: ambulatory healthcare, outpatient healthcare, home healthcare	[1, 2, 6, 9-12, 14, 15, 18, 21, 22]		[8, 17]<br><br>In both studies, parts of data could possibly not have been clearly differentiated from inpatient/institutional care and social services	
**Type of care**: professional medical and nursing care	[1, 2, 8-10, 12, 14, 15, 17, 18, 21, 22]		[6, 11]<br><br>In both studies, parts of data could possibly not have been clearly differentiated from services not provided by healthcare professionals, such as housekeeping	
**CERQual assessment of relevance**	**Minor concerns.** Although the phenomenon of interest in the majority of studies was of indirect relevance (experiences and descriptions instead of preferences and wishes), preferences and wishes were or could be derived from them directly and therefore, we did not have concerns that this negatively impacted the confidence in the review finding. However, in four out of fourteen studies, parts of data could possibly not have been clearly differentiated from institutional care or services not provided by healthcare professionals. Although these studies contributed data to the review finding only to a small extent, we had minor concerns about the impact on confidence.			

**9. Older people value care coordination. [4-6, 8, 10, 12-15, 22]**				
**Dimensions of context**	**Assessment of relevance**			
	**Direct relevance**	**Indirect relevance**	**Partial relevance**	**Uncertain relevance**
**Phenomenon of interest**: Preferences and wishes regarding favorable aspects of ambulatory healthcare	[6, 15]	[4, 5, 8, 10, 12-14, 22]		
**Population/perspective**: people aged 80 and over	[4-6, 8, 10, 12-15, 22]			
**Care setting**: ambulatory healthcare, outpatient healthcare, home healthcare	[4-6, 10, 12-15, 22]		[8]<br><br>Parts of data could possibly not have been clearly differentiated from inpatient/institutional care	
**Type of care**: professional medical and nursing care	[4, 5, 8, 10, 12-15, 22]		[6]<br><br>Parts of data could possibly not have been clearly differentiated from services not provided by healthcare professionals, such as housekeeping	
**CERQual assessment of relevance**	**Minor concerns.** Although the phenomenon of interest in the majority of studies was of indirect relevance (experiences and descriptions instead of preferences and wishes), preferences and wishes were or could be derived from them directly and therefore, we did not have concerns that this negatively impacted the confidence in the review finding. However, in the studies of Modig et al. and Krothe, parts of data could possibly not have been clearly differentiated from institutional care or services not provided by healthcare professionals and this may have affected the strength of the review finding.			

10. **Older people prefer home care. [2, 4, 6, 11, 12, 14, 15, 19, 21]**				
**Dimensions of context**	**Assessment of relevance**			
	**Direct relevance**	**Indirect relevance**	**Partial relevance**	**Uncertain relevance**
**Phenomenon of interest**: Preferences and wishes regarding favorable aspects of ambulatory healthcare	[6, 15]	[2, 4, 11, 12, 14, 19, 21]		
**Population/perspective**: people aged 80 and over	[2, 4, 6, 11, 12, 14, 15, 19, 21]			
**Care setting**: ambulatory healthcare, outpatient healthcare, home healthcare	[2, 4, 6, 11, 12, 14, 15, 19, 21]			
**Type of care**: professional medical and nursing care	[2, 4, 12, 14, 15, 19, 21]		[6, 11]<br><br>In both studies, parts of data could possibly not have been clearly differentiated from services not provided by healthcare professionals, such as housekeeping	
**CERQual assessment of relevance**	**Minor concerns.** Although the phenomenon of interest in the majority of studies was of indirect relevance (experiences and descriptions instead of preferences and wishes), preferences and wishes were or could be derived from them directly and therefore, we did not have concerns that this negatively impacted the confidence in the review finding. In the studies of Krothe and Soodeen et al., parts of data could possibly not have been clearly differentiated from services not provided by healthcare professionals. However, this was subject to only two out of nine studies. Since the study of Krothe contributed a notable part of data to the review finding, we still had minor concerns about the impact on confidence.			

11. **Older people prefer personal information. [1, 8, 15, 17, 19]**				
**Dimensions of context**	**Assessment of relevance**			
	**Direct relevance**	**Indirect relevance**	**Partial relevance**	**Uncertain relevance**
**Phenomenon of interest**: Preferences and wishes regarding favorable aspects of ambulatory healthcare	[1, 15, 17]	[8, 19]		
**Population/perspective**: people aged 80 and over	[1, 8, 15, 17, 19]			
**Care setting**: ambulatory healthcare, outpatient healthcare, home healthcare	[1, 15, 19]		[8, 17]<br><br>In both studies, parts of data could possibly not have been clearly differentiated from inpatient/institutional care and social services	
**Type of care**: professional medical and nursing care	[1, 8, 15, 17, 19]			
**CERQual assessment of relevance**	**Moderate concerns.** Although the phenomenon of interest in two studies was of indirect relevance (experiences and descriptions instead of preferences and wishes), preferences and wishes were or could be derived from them directly and therefore, we did not have concerns that this negatively impacted the confidence in the review finding. However, in two out of five studies, parts of data could possibly not have been clearly differentiated from inpatient/institutional care and social services and this weakened the review finding.			

12. **Older people value advice to help with daily life. [2, 5, 7, 10, 12, 13, 19]**				
**Dimensions of context**	**Assessment of relevance**			
	**Direct relevance**	**Indirect relevance**	**Partial relevance**	**Uncertain relevance**
**Phenomenon of interest**: Preferences and wishes regarding favorable aspects of ambulatory healthcare		[2, 5, 7, 10, 12, 13, 19]		
**Population/perspective**: people aged 80 and over	[2, 5, 7, 10, 12, 13, 19]			
**Care setting**: ambulatory healthcare, outpatient healthcare, home healthcare	[2, 5, 7, 10, 12, 13, 19]			
**Type of care**: professional medical and nursing care	[2, 5, 7, 10, 12, 13, 19]			
**CERQual assessment of relevance**	**No or very minor concerns.** Although the phenomenon of interest in all studies was of indirect relevance (experiences and descriptions instead of preferences and wishes), preferences and wishes were or could be derived from them directly and therefore, we did not have concerns that this negatively impacted the confidence in the review finding.			

13. Older people want information on care options and services. [6, 10, 13, 15, 17, 19]				
**Dimensions of context**	**Assessment of relevance**			
	**Direct relevance**	**Indirect relevance**	**Partial relevance**	**Uncertain relevance**
**Phenomenon of interest**: Preferences and wishes regarding favorable aspects of ambulatory healthcare	[6, 15, 17]	[10, 13, 19]		
**Population/perspective**: people aged 80 and over	[6, 10, 13, 15, 17, 19]			
**Care setting**: ambulatory healthcare, outpatient healthcare, home healthcare	[6, 10, 13, 15, 19]		[17]<br><br>Parts of data could possibly not have been clearly differentiated from inpatient/institutional care and social services	
**Type of care**: professional medical and nursing care	[10, 13, 15, 17, 19]		[6]<br><br>Parts of data could possibly not have been clearly differentiated from services not provided by healthcare professionals, such as housekeeping	
**CERQual assessment of relevance**	**Moderate concerns.** Although the phenomenon of interest half of the studies was of indirect relevance (experiences and descriptions instead of preferences and wishes), preferences and wishes were or could be derived from them directly and therefore, we did not have concerns that this negatively impacted the confidence in the review finding. However, in two out of six studies, parts of data could possibly not have been clearly differentiated from institutional care or services not provided by healthcare professionals and this weakened the review finding.			

14. Older people want to be informed comprehensively. [1, 2, 6, 8-10, 19, 20]

Dimensions of context	Assessment of relevance			
	Direct relevance	Indirect relevance	Partial relevance	Uncertain relevance
**Phenomenon of interest**: Preferences and wishes regarding favorable aspects of ambulatory healthcare	[1, 6, 20]	[2, 8-10, 19]		
**Population/perspective**: people aged 80 and over	[1, 2, 6, 8-10, 19, 20]			
**Care setting**: ambulatory healthcare, outpatient healthcare, home healthcare	[1, 2, 6, 9, 10, 19]		[8, 20]<br><br>In both studies, parts of data could possibly not have been clearly differentiated from inpatient/institutional care	
**Type of care**: professional medical and nursing care	[1, 2, 8-10, 19, 20]		[6]<br><br>Parts of data could possibly not have been clearly differentiated from services not provided by healthcare professionals, such as housekeeping	
**CERQual assessment of relevance**	**Moderate concerns**. Although the phenomenon of interest in the majority of studies was of indirect relevance (experiences and descriptions instead of preferences and wishes), preferences and wishes were or could be derived from them directly and therefore, we did not have concerns that this negatively impacted the confidence in the review finding. However, in three out of eight studies, parts of data could possibly not have been clearly differentiated from institutional care or services not provided by healthcare professionals. Moreover, the study of Modig et al. contributed a large part of data to the review finding, so we had moderate concerns about the impact on confidence.			

15. Older people want more time for their care. [1, 3-5, 8, 9, 14-18]				
**Dimensions of context**	**Assessment of relevance**			
	**Direct relevance**	**Indirect relevance**	**Partial relevance**	**Uncertain relevance**
**Phenomenon of interest**: Preferences and wishes regarding favorable aspects of ambulatory healthcare	[1, 3, 15, 17, 18]	[4, 5, 8, 9, 14, 16]		
**Population/perspective**: people aged 80 and over	[1, 3-5, 8, 9, 14-18]			
**Care setting**: ambulatory healthcare, outpatient healthcare, home healthcare	[1, 3-5, 9, 14, 15, 18]		[8, 17]<br><br>In both studies, parts of data could possibly not have been clearly differentiated from inpatient/institutional care	[16]<br><br>Parts of data could possibly not have been clearly differentiated from inpatient/institutional care
**Type of care**: professional medical and nursing care	[1, 3-5, 8, 9, 14-18]			
**CERQual assessment of relevance**	**Minor concerns.** Although the phenomenon of interest in the majority of studies was of indirect relevance (experiences and descriptions instead of preferences and wishes), preferences and wishes were or could be derived from them directly and therefore, we did not have concerns that this negatively impacted the confidence in the review finding. However, in the studies of Modig et al., Tiilikainen et al. and Schulman-Green et al., parts of data could possibly not have been clearly differentiated from institutional care and the two latter contributed a notable part of data to the review finding. Nevertheless, since these issues affected only three out of thirteen studies, we had minor concerns about the impact on confidence.			

16. Older people expect healthcare professionals to be knowledgeable. [1, 4-6, 8-11, 13, 15, 16, 20]				
**Dimensions of context**	**Assessment of relevance**			
	**Direct relevance**	**Indirect relevance**	**Partial relevance**	**Uncertain relevance**
**Phenomenon of interest**: Preferences and wishes regarding favorable aspects of ambulatory healthcare	[1, 6, 15, 20]	[4, 5, 8-11, 13, 16]		
**Population/perspective**: people aged 80 and over	[1, 4-6, 8-11, 13, 15, 16, 20]			
**Care setting**: ambulatory healthcare, outpatient healthcare, home healthcare	[1, 4-6, 9-11, 13, 15]		[8, 20]<br><br>In both studies, parts of data could possibly not have been clearly differentiated from inpatient/institutional care	[16]<br><br>Parts of data could possibly not have been clearly differentiated from inpatient/institutional care
**Type of care**: professional medical and nursing care	[1, 4, 5, 8-10, 13, 15, 16, 20]		[6, 11]<br><br>In both studies, parts of data could possibly not have been clearly differentiated from services not provided by healthcare professionals, such as housekeeping	
**CERQual assessment of relevance**	**Moderate concerns.** Although the phenomenon of interest in the majority of studies was of indirect relevance (experiences and descriptions instead of preferences and wishes), preferences and wishes were or could be derived from them directly and therefore, we did not have concerns that this negatively impacted the confidence in the review finding. However, in five out of twelve studies, parts of data could possibly not have been clearly differentiated from institutional care or services not provided by healthcare professionals. Moreover, these five studies contributed a notable part of data to the review finding, so we had moderate concerns about the impact on confidence.			

17. Older people value healthcare professionals' communication skills. [5, 10-13, 15, 20]				
**Dimensions of context**	**Assessment of relevance**			
	**Direct relevance**	**Indirect relevance**	**Partial relevance**	**Uncertain relevance**
**Phenomenon of interest**: Preferences and wishes regarding favorable aspects of ambulatory healthcare	[15, 20]	[5, 10-13]		
**Population/perspective**: people aged 80 and over	[5, 10-13, 15, 20]			
**Care setting**: ambulatory healthcare, outpatient healthcare, home healthcare	[5, 10-13, 15]		[20]<br><br>Parts of data could possibly not have been clearly differentiated from inpatient/institutional care	
**Type of care**: professional medical and nursing care	[5, 10, 12, 13, 15, 20]		[11]<br><br>Parts of data could possibly not have been clearly differentiated from services not provided by healthcare professionals, such as housekeeping	
**CERQual assessment of relevance**	**No or very minor concerns.** Although the phenomenon of interest in the majority of studies was of indirect relevance (experiences and descriptions instead of preferences and wishes), preferences and wishes were or could be derived from them directly and therefore, we did not have concerns that this negatively impacted the confidence in the review finding. In two out of seven studies, parts of data could possibly not have been clearly differentiated from institutional care or services provided by healthcare professionals. However, these two studies contributed only a very small part of data to the review finding, so we had only very minor concerns about the impact on confidence.			

**18. Older people wish to receive personal attention. [1-3, 5-7, 9-14, 17, 19, 21, 22]**				
**Dimensions of context**	**Assessment of relevance**			
	**Direct relevance**	**Indirect relevance**	**Partial relevance**	**Uncertain relevance**
**Phenomenon of interest**: Preferences and wishes regarding favorable aspects of ambulatory healthcare	[1, 3, 6, 17]	[2, 5, 7, 9-14, 19, 21, 22]		
**Population/perspective**: people aged 80 and over	[1-3, 5-7, 9-14, 17, 19, 21, 22]			
**Care setting**: ambulatory healthcare, outpatient healthcare, home healthcare	[1-3, 5-7, 9-14, 19, 21, 22]		[17]<br><br>Parts of data could possibly not have been clearly differentiated from inpatient/institutional care	
**Type of care**: professional medical and nursing care	[1-3, 5, 7, 9, 10, 12-14, 17, 19, 21, 22]		[6, 11]<br><br>In both studies, parts of data could possibly not have been clearly differentiated from services not provided by healthcare professionals, such as housekeeping	
**CERQual assessment of relevance**	**No or very minor concerns.** Although the phenomenon of interest in the majority of studies was of indirect relevance (experiences and descriptions instead of preferences and wishes), preferences and wishes were or could be derived from them directly and therefore, we did not have concerns that this negatively impacted the confidence in the review finding. In the studies of Krothe et al., Soodeen et al., and Tiilikainen et al., parts of data could possibly not have been clearly differentiated from institutional care or services not provided by healthcare professionals. However, this was subject to only three out of sixteen studies and the three studies in question contributed only a very small part of data to the review finding.			

19. Older people value close, long-term relationships. [1, 2, 5-7, 9-11, 14, 18, 21, 22]				
**Dimensions of context**	**Assessment of relevance**			
	**Direct relevance**	**Indirect relevance**	**Partial relevance**	**Uncertain relevance**
**Phenomenon of interest**: Preferences and wishes regarding favorable aspects of ambulatory healthcare	[1, 6, 18]	[2, 5, 7, 9-11, 14, 21, 22]		
**Population/perspective**: people aged 80 and over	[1, 2, 5-7, 9-11, 14, 18, 21, 22]			
**Care setting**: ambulatory healthcare, outpatient healthcare, home healthcare	[1, 2, 5-7, 9-11, 14, 18, 21, 22]			
**Type of care**: professional medical and nursing care	[1, 2, 5, 7, 9, 10, 14, 18, 21, 22]		[6, 11]<br><br>In both studies, parts of data could possibly not have been clearly differentiated from services not provided by healthcare professionals, such as housekeeping	
**CERQual assessment of relevance**	**No or very minor concerns**. Although the phenomenon of interest in the majority of studies was of indirect relevance (experiences and descriptions instead of preferences and wishes), preferences and wishes were or could be derived from them directly and therefore, we did not have concerns that this negatively impacted the confidence in the review finding. In the studies of Krothe et al. and Soodeen et al., parts of data could possibly not have been clearly differentiated from services not provided by healthcare professionals. However, this was subject to only two out of twelve studies.			

20. Older people want to be treated in a friendly way. [1-3, 5, 7, 9-11, 13, 17, 21]				
**Dimensions of context**	**Assessment of relevance**			
	**Direct relevance**	**Indirect relevance**	**Partial relevance**	**Uncertain relevance**
**Phenomenon of interest**: Preferences and wishes regarding favorable aspects of ambulatory healthcare	[1, 3, 17]	[2, 5, 7, 9-11, 13, 21]		
**Population/perspective**: people aged 80 and over	[1-3, 5, 7, 9-11, 13, 17, 21]			
**Care setting**: ambulatory healthcare, outpatient healthcare, home healthcare	[1-3, 5, 7, 9-11, 13, 21]		[17]<br><br>Parts of data could possibly not have been clearly differentiated from inpatient/institutional care	
**Type of care**: professional medical and nursing care	[1-3, 5, 7, 9, 10, 13, 17, 21]		[11]<br><br>Parts of data could possibly not have been clearly differentiated from services not provided by healthcare professionals, such as housekeeping	
**CERQual assessment of relevance**	**Minor concerns.** Although the phenomenon of interest in the majority of studies was of indirect relevance (experiences and descriptions instead of preferences and wishes), preferences and wishes were or could be derived from them directly and therefore, we did not have concerns that this negatively impacted the confidence in the review finding. However, in the studies of Soodeen et al., and Tiilikainen et al., parts of data could possibly not have been clearly differentiated from institutional care or services not provided by healthcare professionals and this may have affected the strength of the review finding.			

21. Older people value open and confidential communication. [1, 5, 6, 8-15, 18, 21]				
**Dimensions of context**	**Assessment of relevance**			
	**Direct relevance**	**Indirect relevance**	**Partial relevance**	**Uncertain relevance**
**Phenomenon of interest**: Preferences and wishes regarding favorable aspects of ambulatory healthcare	[1, 6, 15, 18]	[5, 8-14, 21]		
**Population/perspective**: people aged 80 and over	[1, 5, 6, 8-15, 18, 21]			
**Care setting**: ambulatory healthcare, outpatient healthcare, home healthcare	[1, 5, 6, 9-15, 18, 21]		[8]<br><br>Parts of data could possibly not have been clearly differentiated from inpatient/institutional care	
**Type of care**: professional medical and nursing care	[1, 5, 8-10, 12-15, 18, 21]		[6, 11]<br><br>In both studies, parts of data could possibly not have been clearly differentiated from services not provided by healthcare professionals, such as housekeeping	
**CERQual assessment of relevance**	**No or very minor concerns**. Although the phenomenon of interest in the majority of studies was of indirect relevance (experiences and descriptions instead of preferences and wishes), preferences and wishes were or could be derived from them directly and therefore, we did not have concerns that this negatively impacted the confidence in the review finding. In the studies of Modig et al., Krothe and Soodeen et al., parts of data could possibly not have been clearly differentiated from institutional care or services not provided by healthcare professionals. However, this was subject to only three out of thirteen studies and the three studies in question contributed only a very small part of data to the review finding.			

**22. Older people want to be involved in decisions and care. [1, 2, 4-6, 8, 9, 11, 12, 14-18, 21, 22]**				
**Dimensions of context**	**Assessment of relevance**			
	**Direct relevance**	**Indirect relevance**	**Partial relevance**	**Uncertain relevance**
**Phenomenon of interest**: Preferences and wishes regarding favorable aspects of ambulatory healthcare	[1, 6, 15, 17, 18]	[2, 4, 5, 8, 9, 11, 12, 14, 16, 21, 22]		
**Population/perspective**: people aged 80 and over	[1, 2, 4-6, 8, 9, 11, 12, 14-18, 21, 22]			
**Care setting**: ambulatory healthcare, outpatient healthcare, home healthcare	[1, 2, 4-6, 9, 11, 12, 14, 15, 18, 21, 22]		[8, 17]<br><br>In both studies, parts of data could possibly not have been clearly differentiated from inpatient/institutional care	[16]<br><br>Parts of data could possibly not have been clearly differentiated from inpatient/institutional care
**Type of care**: professional medical and nursing care	[1, 2, 4, 5, 8, 9, 12, 14-18, 21, 22]		[6, 11]<br><br>In both studies, parts of data could possibly not have been clearly differentiated from services not provided by healthcare professionals, such as housekeeping	
**CERQual assessment of relevance**	**Minor concerns.** Although the phenomenon of interest studied in the majority of studies was of indirect relevance (experiences and descriptions instead of preferences and wishes), preferences and wishes were or could be derived from them directly and therefore, we did not have concerns that this negatively impacted the confidence in the review finding. In the studies of Modig et al., Tiilikainen et al., Schulman-Green et al., Krothe and Soodeen et al., parts of data could possibly not have been clearly differentiated from services not provided by healthcare professionals or in institutional settings. However, this was subject to only four out of sixteen studies and the studies in question contributed only a relatively small part of data to the review finding, so we had minor concerns about the impact on confidence.			

**23. Older people value activity. [3, 4, 6, 9, 10, 12-15, 19, 20, 22]**				
**Dimensions of context**	**Assessment of relevance**			
	**Direct relevance**	**Indirect relevance**	**Partial relevance**	**Uncertain relevance**
**Phenomenon of interest**: Preferences and wishes regarding favorable aspects of ambulatory healthcare	[3, 6, 15, 20]	[4, 9, 10, 12-14, 19, 22]		
**Population/perspective**: people aged 80 and over	[3, 4, 6, 9, 10, 12-15, 19, 20, 22]			
**Care setting**: ambulatory healthcare, outpatient healthcare, home healthcare	[3, 4, 6, 9, 10, 12-15, 19, 22]		[20]<br><br>Parts of data could possibly not have been clearly differentiated from inpatient/institutional care	
**Type of care**: professional medical and nursing care	[3, 4, 9, 10, 12-15, 19, 20, 22]		[6]<br><br>Parts of data could possibly not have been clearly differentiated from services not provided by healthcare professionals, such as housekeeping	
**CERQual assessment of relevance**	**No or very minor concerns.** Although the phenomenon of interest in the majority of studies was of indirect relevance (experiences and descriptions instead of preferences and wishes), preferences and wishes were or could be derived from them directly and therefore, we did not have concerns that this negatively impacted the confidence in the review finding. In the studies of Walker et al. und Krothe, parts of data could possibly not have been clearly differentiated from institutional care or services not provided by healthcare professionals. However, this was subject to only two out of twelve studies and the two studies in question contributed only a very small part of data to the review finding.			

References

1. Berkelmans PG, Berendsen AJ, Verhaak PF, van der Meer K. Characteristics of general practice care: what do senior citizens value? A qualitative study. BMC Geriatr. 2010;10:80. https://doi.org/10.1186/1471-2318-10-80.

2. Bjornsdottir K. 'Holding on to life': An ethnographic study of living well at home in old age. Nurs Inq. 2018;25(2):1. https://doi.org/10.1111/nin.12228.

3. Faeo SE, Bruvik FK, Tranvag O, Husebo BS. Home-dwelling persons with dementia's perception on care support: Qualitative study. Nurs Ethics. 2020. https://doi.org/10.1177/0969733019893098.

4. Gowing A, Dickinson C, Gorman T, Robinson L, Duncan R. Patients' experiences of a multidisciplinary team-led community case management programme: a qualitative study. BMJ Open. 2016;6(9):e012019. https://doi.org/10.1136/bmjopen-2016-012019.

5. King AII, Boyd ML, Dagley L, Raphael DL. Implementation of a gerontology nurse specialist role in primary health care: Health professional and older adult perspectives. J Clin Nurs. 2018;27(3-4):807-18. https://doi.org/10.1111/jocn.14110.

6. Krothe JS. Constructions of elderly people's perceived needs for community-based long-term care. Indiana University School of Nursing; 1992.

7. Michel T, Helena Lenardt M, Hautsch Willig M, Maria Alvarez A. From real to ideal - the health (un)care of long-lived elders. Rev Bras Enferm. 2015;68(3):343-9. https://doi.org/10.1590/0034-7167.2015680304i.

8. Modig S, Kristensson J, Troein M, Brorsson A, Midlöv P. Frail elderly patients' experiences of information on medication. A qualitative study. BMC Geriatr. 2012;12(1):46. https://doi.org/10.1186/1471-2318-12-46.

9. Moe A, Hellzen O, Enmarker I. The meaning of receiving help from home nursing care. Nurs Ethics. 2013;20(7):737-47. https://doi.org/10.1177/0969733013478959.

10. Sandberg M, Jakobsson U, Midlov P, Kristensson J. Case management for frail older people - a qualitative study of receivers' and providers' experiences of a complex intervention. BMC Health Serv Res. 2014;14. https://doi.org/10.1186/1472-6963-14-14.

11. Soodeen RA, Gregory D, Bond JB. Home care for older couples: "It feels like a security blanket.. ". Qual Health Res. 2007;17(9):1245-55. https://doi.org/10.1177/1049732307307339.

12. Spoorenberg SLW, Wynia K, Fokkens AS, Slotman K, Kremer HPH, Reijneveld SA. Experiences of Community-Living Older Adults Receiving Integrated Care Based on the Chronic Care Model: A Qualitative Study. PLoS One. 2015;10(10):1. https://doi.org/10.1371/journal.pone.0137803.

13. Toien M, Bjork IT, Fagerstrom L. Older users' perspectives on the benefits of preventive home visits. Qual Health Res. 2015;25(5):700-12. https://doi.org/10.1177/1049732314553595.

14. Turjamaa R, Hartikainen S, Kangasniemi M, Pietila AM. Living longer at home: a qualitative study of older clients' and practical nurses' perceptions of home care. J Clin Nurs. 2014;23(21-22):3206-17. https://doi.org/10.1111/jocn.12569.

15. van Blijswijk SCE, de Waard CS, van Peet PG, Keizer D, von Faber M, de Waal MWM, et al. Wishes and needs of community-dwelling older persons concerning general practice: A qualitative study. PLoS One. 2018;13(7):14. https://doi.org/10.1371/journal.pone.0200614.

16. Schulman-Green DJ, Naik AD, Bradley EH, McCorkle R, Bogardus ST. Goal setting as a shared decision making strategy among clinicians and their older patients. Patient Educ Couns. 2006;63(1-2):145-51. https://doi.org/10.1016/j.pec.2005.09.010.

17. Tiilikainen E, Hujala A, Kannasoja S, Rissanen S, Närhi K. "They're always in a hurry" – Older people´s perceptions of access and recognition in health and social care services. Health Soc Care Community. 2019;27(4):1011-8. https://doi.org/10.1111/hsc.12718.

18. van Kempen JA, Robben SH, Zuidema SU, Rikkert MG, Melis RJ, Schers HJ. Home visits for frail older people: a qualitative study on the needs and preferences of frail older people and their informal caregivers. Br J Gen Pract. 2012;62(601):554-60. https://doi.org/10.3399/bjgp12X653606.

19. Behm L, Ivanoff SD, Ziden L. Preventive home visits and health: experiences among very old people. BMC Public Health. 2013;13:378. https://doi.org/10.1186/1471-2458-13-378.

20. Walker R, Ratcliffe J, White A, Visvanathan R. Dementia assessment services: What are the perceptions of older people? Australas J Ageing. 2018;37(1):43-7. https://doi.org/10.1111/ajag.12455.

21. Jarling A, Rydstrom I, Ernsth-Bravell M, Nystrom M, Dalheim-Englund AC. Becoming a guest in your own home: Home care in Sweden from the perspective of older people with multimorbidities. Int J Older People Nurs. 2018;13(3). https://doi.org/10.1111/opn.12194.

22. Martin-Matthews A, Sims-Gould J. Employers, home support workers and elderly clients: identifying key issues in delivery and receipt of home support. Healthc Q. 2008;11(4):69-75. https://doi.org/10.12927/hcq.2008.20073.
