# Supplementary material for: Which features of ambulatory healthcare are preferred by people aged 80 and over? Findings from a systematic review of qualitative studies and appraisal of confidence using GRADE-CERQual
Source: BMC Geriatr. 2022 May 16;22:428. doi: 10.1186/s12877-022-03006-6 (PMC9109291; doi:10.1186/s12877-022-03006-6)
Supplement: Supplementary file 4 — Additional file 4. CERQual evidence profile. [file 12877_2022_3006_MOESM4_ESM.pdf]

Herrler A, Kukla H, Vennedey V, Stock S. Which features of ambulatory healthcare are preferred by people aged 80 and over? Findings from a systematic review of qualitative studies and appraisal of confidence using GRADE-CERQual. BMC Geriatrics.

Corresponding author: Angélique Herrler, Faculty of Human Sciences and Faculty of Medicine, Graduate School GROW – Gerontological Research on Well-being, University of Cologne, Albertus-Magnus-Platz, 50923 Cologne, Germany; e-mail: angelique.herrler@uni-koeln.de

Additional file 4: CERQual evidence profile

Summary of review finding	Studies contributing to the review finding	Methodological limitations	Coherence	Adequacy	Relevance	Confidence in the evidence	Explanation of CERQual assessment
**1. Older people wish to receive care that fits their individual needs.**	[1-17]	**No or very minor concerns**<br>The study of King et al. was less trustworthy due to a very one-sided, positive evaluation of the intervention without discussion of the researcher's position. The analysis of Bjornsdottir did not report on discrepant results and was conducted by only one researcher. We had very minor concerns, because these limitations related to only two of seventeen studies and therefore, did not affect the review finding notably.	**Minor concerns**<br>While approximately two thirds of underlying data directly supported this review finding, one third rather indirectly described the core of the finding or did not explore it in detail. However, there were no contradictory data.	**No or very minor concerns**<br>Seventeen studies contributed to this review finding and represented a variety of examples that illustrated or explained the finding. Although some findings did not directly describe the review finding (see coherence), the other findings still provided rich underlying data.	**Minor concerns**<br>Although the phenomenon of interest in the majority of studies was of indirect relevance (experiences and descriptions instead of preferences and wishes), preferences and wishes were or could be derived from them directly and therefore, we did not have concerns that this negatively impacted the confidence in the review finding. However, in five out of sixteen studies, parts of data could possibly not have been clearly differentiated from services not provided by healthcare professionals or in institutional settings. Since these studies only represented a small part of contributing data, our concerns regarding the	**High**	Seventeen studies with no or very minor concerns regarding methodological limitations and adequacy contributed to this review finding. Although there were minor concerns about coherence and relevance, this was only due to a limited number of studies/extent of data.

Summary of review finding	Studies contributing to the review finding	Methodological limitations	Coherence	Adequacy	Relevance	Confidence in the evidence	Explanation of CERQual assessment
					impact on relevance of this review finding remained minor.		
**2. Older people value being looked after regularly.**	[2-5, 8, 10, 12-15]	**Minor concerns**<br><br>The study of King et al. was less trustworthy due to a very one-sided, positive evaluation of the intervention without discussion of the researcher's position. The analysis of Bjornsdottir did not report on discrepant results and was conducted by only one researcher. We had minor concerns, because these limitations related to two of ten studies and weakened the review finding to a limited extent.	**Minor concerns**<br><br>While approximately two thirds of underlying data directly supported this review finding, one third rather indirectly described the core of the finding or did not explore it in detail. However, there were no contradictory data.	**No or very minor concerns**<br><br>Ten studies contributed to this review finding and represented a variety of examples that illustrate or explained the finding. Although some findings did not directly describe the review finding, the other findings still provided rich underlying data.	**No or very minor concerns**<br><br>Although the phenomenon of interest in the majority of studies was of indirect relevance (experiences and descriptions instead of preferences and wishes), preferences and wishes were or could be derived from them directly. The fact that parts of data could possibly not have been clearly differentiated from services not provided by healthcare professionals or in institutional settings was only subject to one out of ten studies. Moreover, this study contributed only a very small part of data to the review finding. Therefore, we did not have concerns that there were negative impacts on the confidence in this review finding.	**High**	Ten studies with no or very minor concerns regarding adequacy and relevance contributed to this review finding. Although there were minor concerns about methodological limitations and coherence, this was only due to a limited number of studies/extent of data.
**3. Older people accept delegation.**	[1, 4, 15, 17, 18]	**No or very minor concerns**<br><br>None of the studies contributing to this review finding were found to have	**Moderate concerns**<br><br>Most underlying data directly referred to this review finding and the studies in question	**Moderate concerns**<br><br>Only five studies contributed to this review finding and some of them reported different views	**Minor concerns**<br><br>Although the phenomenon of interest studied in Gowing et al. was of indirect relevance	**Low**	Five studies contributed to this review finding. While there were no or very minor concerns regarding methodological limitations, there were moderate

Summary of review finding	Studies contributing to the review finding	Methodological limitations	Coherence	Adequacy	Relevance	Confidence in the evidence	Explanation of CERQual assessment
		notable methodological limitations.	reported that the majority of their participants accepted delegation. However, this was subject to certain conditions and three out of five studies also reported deviating opinions of their participants.	regarding delegation. Although studies described that "some" or "most" of their participants represented the according views, this was not clearly quantifiable. However, the underlying data delivered sufficient explanation to the core of this this review finding.	(experiences and descriptions instead of preferences and wishes), preferences and wishes were or could be derived from them directly. However, in another study, parts of data could possibly not have been clearly differentiated from services not provided by healthcare professionals or in institutional settings. Although this study contributed only a small part of data to this review finding, two out of five studies were affected by indirect or partial relevance, so we had minor concerns that this negatively impacted the confidence in this review finding.		concerns regarding coherence and adequacy because of the small number of studies and partially contradictory data. Moreover, there were minor concerns about relevance.
**4. Older people value home visits, but not all think they are necessary.**	[1, 15, 18] [19]	**No or very minor concerns**<br><br>None of the studies contributing to this review finding were found to have notable methodological limitations.	**Moderate concerns**<br><br>Most underlying data directly referred to this review finding. However, it was not clear in which cases home visits were only a useful "add-on" to their healthcare and in which they were really necessary. We described this as moderate instead of serious concern, because the conditions and variety of opinions in	**Moderate concerns**<br><br>Only four studies contributed to this review findings. Although the underlying data sufficiently explained the different motives in the valuation of home visits, the primary studies' did not quantify the participants representing the according views.	**No or very minor concerns**<br><br>Although the phenomenon of interest in one out of four studies was of indirect relevance (experiences and descriptions instead of preferences and wishes), preferences and wishes were or could be derived directly and therefore, we did not have concerns that this negatively impacted	**Low**	Four studies contributed to this review finding. While there were no or very minor concerns regarding methodological limitations and relevance, there were moderate concerns regarding coherence and adequacy because of the small number of studies and partially contradictory data.

Summary of review finding	Studies contributing to the review finding	Methodological limitations	Coherence	Adequacy	Relevance	Confidence in the evidence	Explanation of CERQual assessment
			general were part of the review finding's description and already referred to the ambiguous data.		the confidence in the review finding.		
**5. Older people want fast contact to care.**	[1, 4, 8, 9, 12, 13, 15, 19]	**No or very minor concerns**<br><br>None of the studies contributing to this review finding were found to have notable methodological limitations.	**No or very minor concerns**<br><br>The vast majority of underlying data directly and unambiguously supported the review finding and there were no contradictory data.	**No or very minor concerns**<br><br>Eight studies contributed to this review finding and most of their findings also included rich explanations about why older people wanted fast contact to care.	**No or very minor concerns**<br><br>Although the phenomenon of interest in the majority of studies was of indirect relevance (experiences and descriptions instead of preferences and wishes), preferences and wishes were or could be derived from them directly and therefore, we did not have concerns that this negatively impacted the confidence in the review finding. In the study of Modig et al., parts of data could possibly not have been clearly differentiated from services not provided by healthcare professionals or in institutional settings. However, this was subject to only one out of eight studies and the contribution of data from this study was very small.	**High**	Eight studies contributed to this review finding. There were no or very minor concerns regarding methodological limitations, coherence, adequacy and relevance.
**6. Older people want easy access to care.**	[1, 2, 5, 6, 8, 12, 14, 15, 17, 20]	**No or very minor concerns**<br><br>The study of King et al. was less trustworthy due to a very	**No or very minor concerns**	**No or very minor concerns**	**Moderate concerns**<br><br>Although the phenomenon of interest in	**High**	Ten studies contributed to this review finding. There were no or very minor concerns regarding

Summary of review finding	Studies contributing to the review finding	Methodological limitations	Coherence	Adequacy	Relevance	Confidence in the evidence	Explanation of CERQual assessment
		one-sided, positive evaluation of the intervention without discussion of the researcher's position. The analysis of Bjornsdottir did not report on discrepant results and was conducted by only one researcher. We had very minor concerns, because these limitations related to two of ten studies, the studies contributed to the review finding only to a small extent and therefore, did not affect the review finding notably.	The vast majority of underlying data directly and unambiguously supported the review finding and there were no contradictory data. The meaning of "access" differed between the primary studies sometimes, but this was considered in the review finding's description.	Ten studies contributed to this review finding and most of their findings included an explanation what easy access meant to the participants.	approximately half of the studies was of indirect relevance (experiences and descriptions instead of preferences and wishes), preferences and wishes were or could be derived from them directly and therefore, we did not have concerns that this negatively impacted the confidence in the review finding. However, in four out of ten studies, parts of data could possibly not have been clearly differentiated from institutional care or services not provided by healthcare professionals. Since two of them (Tiilikainen et al. and Walker et al.) contributed a notable part of data to this review finding, we had moderate concerns about the impact on confidence.		methodological limitations, coherence and adequacy. Although there were moderate concerns regarding relevance, the review finding still is a valid representation of the data.
**7. Older people reject waiting times.**	[1, 2, 8, 21]	**No or very minor concerns**<br><br>The analysis of Bjornsdottir did not report on discrepant results and was conducted by only one researcher. We had very minor concerns, because this limitation related to only one of four studies, the study contributed to the review finding only to a very small	**No or very minor concerns**<br><br>The underlying data directly and unambiguously supported the review finding and there were no contradictory data.	**Minor concerns**<br><br>Only four studies contributed to this review finding, but these studies provided a variety of examples and descriptions on the negative perception of waiting times.	**Minor concerns**<br><br>Although the phenomenon of interest in the majority of studies was of indirect relevance (experiences and descriptions instead of preferences and wishes), preferences and wishes were or could be derived	**Moderate**	Four studies contributed to this review finding. There were no or very minor concerns regarding methodological limitations and coherence. However, there were minor concerns regarding adequacy and relevance and due to the quite small number of

Summary of review finding	Studies contributing to the review finding	Methodological limitations	Coherence	Adequacy	Relevance	Confidence in the evidence	Explanation of CERQual assessment
		extent and therefore, did not affect the review finding notably.			from them directly and therefore, we did not have concerns that this negatively impacted the confidence in the review finding. However, in the study of Modig et al., parts of data could possibly not have been clearly differentiated from services not provided by healthcare professionals or in institutional settings and this may have affected the strength of the review finding.		studies, we found that this weakened the review finding.
**8. Older people want reliable and continuous care.**	[1, 2, 6, 8-12, 14, 15, 17, 18, 21, 22]	**No or very minor concerns**<br><br>The analysis of Bjornsdottir did not report on discrepant results and was conducted by only one researcher. The study of Martin-Matthews et al. did not report on ethical approval, but the study's aim and results did not seem to be affected by that. We had very minor concerns, because these limitations related to two of fourteen studies, the studies contributed to the review finding only to a small extent and therefore, did not affect the review finding notably.	**No or very minor concerns**<br><br>The vast majority of underlying data directly and unambiguously supported the review finding and there were no contradictory data.	**No or very minor concerns**<br><br>Fourteen studies contributed to this review finding and represented a variety of examples that illustrated or explained the finding.	**Minor concerns**<br><br>Although the phenomenon of interest in the majority of studies was of indirect relevance (experiences and descriptions instead of preferences and wishes), preferences and wishes were or could be derived from them directly and therefore, we did not have concerns that this negatively impacted the confidence in the review finding. However, in four out of fourteen studies, parts of data could possibly not have been clearly differentiated from institutional care or services not provided by	**High**	Fourteen studies contributed to this review finding. There were no or very minor concerns regarding methodological limitations, coherence and adequacy. Although there were minor concerns regarding relevance, there was in sum no negative impact on the review finding.

Summary of review finding	Studies contributing to the review finding	Methodological limitations	Coherence	Adequacy	Relevance	Confidence in the evidence	Explanation of CERQual assessment
					healthcare professionals. Although these studies contributed data to the review finding only to a small extent, we had minor concerns about the impact on confidence.		
**9. Older people value care coordination.**	[4-6, 8, 10, 12-15, 22]	**Moderate concerns**<br><br>The study of King et al. was less trustworthy due to a very one-sided, positive evaluation of the intervention without discussion of the researcher's position. The study of Martin-Matthews et al. did not report on ethical approval, but the study's aim and results did not seem to be affected by that. We had moderate concerns, because these limitations related to two of ten studies and while the study of Martin-Matthews and Sims-Gould contributed to the review finding only to a very small extent, the limitations in the study of King et al. weakened the confidence in this review finding.	**No or very minor concerns**<br><br>Although only half of the underlying data directly supported the review finding, the other half did so by describing the wish for a person that helped them manage their issues and there were no contradictory data.	**No or very minor concerns**<br><br>Ten studies contributed to this review finding and represented a variety of examples that illustrated or explained the finding.	**Minor concerns**<br><br>Although the phenomenon of interest in the majority of studies was of indirect relevance (experiences and descriptions instead of preferences and wishes), preferences and wishes were or could be derived from them directly and therefore, we did not have concerns that this negatively impacted the confidence in the review finding. However, in the studies of Modig et al. and Krothe, parts of data could possibly not have been clearly differentiated from institutional care or services not provided by healthcare professionals and this may have affected the strength of the review finding.	**Moderate**	Ten studies contributed to this review finding. There were no or very minor concerns regarding coherence and adequacy. However, there were moderate concerns regarding methodological limitations and relevance that weakened the review finding in total.
**10. Older people prefer home care.**	[2, 4, 6, 11, 12, 14, 15, 19, 21]	**No or very minor concerns**<br><br>The analysis of Bjornsdottir did not report on discrepant results and was only	**Minor concerns**<br><br>The vast majority of underlying data directly and unambiguously	**No or very minor concerns**<br><br>Nine studies contributed to this review finding and	**Minor concerns**<br><br>Although the phenomenon of interest in the majority of studies	**High**	Nine studies contributed to this review finding. There were no or very minor concerns regarding methodological limitations

Summary of review finding	Studies contributing to the review finding	Methodological limitations	Coherence	Adequacy	Relevance	Confidence in the evidence	Explanation of CERQual assessment
		conducted by only one researcher. We had very minor concerns, because this limitation related to only one of nine studies, the study contributed to the review finding only to a small extent and therefore, did not affect the review finding notably.	supported the review finding. However, in the study of Gowing et al., it was described that some participants preferred the hospital under certain conditions.	represented a variety of examples that illustrated or explained the finding.	was of indirect relevance (experiences and descriptions instead of preferences and wishes), preferences and wishes were or could be derived from them directly and therefore, we did not have concerns that this negatively impacted the confidence in the review finding. In the studies of Krothe and Soodeen et al., parts of data could possibly not have been clearly differentiated from services not provided by healthcare professionals. However, this was subject to only two out of nine studies. Since the study of Krothe contributed a notable part of data to the review finding, we still had minor concerns about the impact on confidence.		and adequacy. Although there were minor concerns regarding coherence and relevance, this did not significantly affect the review finding, which was still a valid representation of the data.
**11. Older people prefer personal information.**	[1, 8, 15, 17, 19]	**No or very minor concerns**<br><br>None of the studies contributing to this review finding were found to have notable methodological limitations.	**Minor concerns**<br><br>Although the majority of underlying data directly supported the review finding, two studies suggested that written information can be an important, too. However, this was described as a supplementary to support personally communicated information.	**Minor concerns**<br><br>Minor concerns. Only five studies contributed to this review finding but they provided a variety of examples and descriptions on the advantage of personal information.	**Moderate concerns**<br><br>Although the phenomenon of interest in two studies was of indirect relevance (experiences and descriptions instead of preferences and wishes), preferences and wishes were or could be derived from them directly and therefore, we did not have	**Low**	Five studies contributed to this review finding. While there were no or very minor concerns regarding methodological limitations, there were moderate concerns regarding relevance. Moreover, there were minor concerns regarding coherence and adequacy. Since the number of contributing studies was small, we found that this

**Summary of review finding**	**Studies contributing to the review finding**	**Methodological limitations**	**Coherence**	**Adequacy**	**Relevance**	**Confidence in the evidence**	**Explanation of CERQual assessment**
					concerns that this negatively impacted the confidence in the review finding. However, in two out of five studies, parts of data could possibly not have been clearly differentiated from inpatient/institutional care and social services and this weakened the review finding.		significantly impacted the strength of the review finding.
**12. Older people value advice to help with daily life.**	[2, 5, 7, 10, 12, 13, 19]	**Moderate concerns**<br><br>The analysis of Bjornsdottir did not report discrepant results and was conducted by only one researcher. The study of King et al. was less trustworthy due to a very one-sided, positive evaluation of the intervention without discussion of the researcher's position. We had moderate concerns because these limitations related to two of seven studies and while the study of Bjornsdottir contributed to the review finding only to a very small extent, the limitations in the study of King et al. weakened the confidence in this review finding.	**No or very minor concerns**<br><br>The vast majority of underlying data directly and unambiguously supported the review finding and there were no contradictory data.	**No or very minor concerns**<br><br>Seven studies contributed to this review finding and represented a variety of examples that illustrated or explained the finding.	**No or very minor concerns**<br><br>Although the phenomenon of interest in all studies was of indirect relevance (experiences and descriptions instead of preferences and wishes), preferences and wishes were or could be derived from them directly and therefore, we did not have concerns that this negatively impacted the confidence in the review finding.	**High**	Seven studies contributed to this review finding. There were no or very minor concerns regarding coherence, adequacy and relevance. However, there were moderate concerns regarding methodological limitations. Since this is mostly due to one study, there was no significant impact on the review finding in total.
**13. Older people want information on**	[6, 10, 13, 15, 17, 19]	**No or very minor concerns**<br><br>None of the studies contributing to this review finding were found to have	**Minor concerns**<br><br>While approximately the half of underlying data directly supported this	**Minor concerns**<br><br>Only six studies contributed to this review finding but the primary	**Moderate concerns**<br><br>Although the phenomenon of interest half of the studies was of	**Moderate**	Six studies contributed to this review finding. While there were no or very minor concerns regarding methodological limitations,

Summary of review finding	Studies contributing to the review finding	Methodological limitations	Coherence	Adequacy	Relevance	Confidence in the evidence	Explanation of CERQual assessment
**care options and services.**		notable methodological limitations.	review finding, the other half did so rather indirectly. However, there were no contradictory data and the indirectly supporting half of data also illustrated examples in favor of the review finding.	studies provided a variety of examples and descriptions on the usefulness and desirability of information on care options and services.	indirect relevance (experiences and descriptions instead of preferences and wishes), preferences and wishes were or could be derived from them directly and therefore, we did not have concerns that this negatively impacted the confidence in the review finding. However, in two out of six studies, parts of data could possibly not have been clearly differentiated from institutional care or services not provided by healthcare professionals and this weakened the review finding.		there were minor concerns regarding coherence and adequacy. Moreover, there were moderate concerns regarding relevance that weakened the review finding.
**14. Older people want to be informed comprehensively.**	[1, 2, 6, 8-10, 19, 20]	**No or very minor concerns**<br><br>The analysis of Bjornsdottir did not report on discrepant results and was conducted by only one researcher. We had very minor concerns because this limitation related to only one of eight studies, the study contributed to this review finding only to a small extent and therefore, did not affect the review finding notably.	**Moderate concerns**<br><br>Only half of the underlying data directly referred to the wish of being informed comprehensively. Moreover, within some of the studies, it was described that some older people were satisfied with limited information or scared by too much information. However, we described this as moderate instead of serious concern, because the conditions and variety of	**Moderate concerns**<br><br>Eight studies contributed to this review finding and represented a variety of examples and descriptions about the wish to receive comprehensive information. However, the study of Modig et al. contributed the largest part of data to this finding and the other contributing studies did not provide the same level of data richness as Modig et al.	**Moderate concerns**<br><br>Although the phenomenon of interest in the majority of studies was of indirect relevance (experiences and descriptions instead of preferences and wishes), preferences and wishes were or could be derived from them directly and therefore, we did not have concerns that this negatively impacted the confidence in the review finding. However, in three out of eight studies, parts	**Low**	Eight studies contributed to this review finding. There were no or very minor concerns regarding methodological limitations. However, there were moderate concerns regarding coherence, adequacy and relevance. Since there was one study with limitations that provided a large part of data and moreover, there were partially contradictory data, there was a strong weakening of the review finding.

Summary of review finding	Studies contributing to the review finding	Methodological limitations	Coherence	Adequacy	Relevance	Confidence in the evidence	Explanation of CERQual assessment
			opinions in general were part of the review finding's description and already referred to the ambiguous data.		of data could possibly not have been clearly differentiated from institutional care or services not provided by healthcare professionals. Moreover, the study of Modig et al. contributed a large part of data to the review finding, so we had moderate concerns about the impact on confidence.		
**15. Older people want more time for their care.**	[1, 3-5, 8, 9, 14-18]	**No or very minor concerns**<br>The study of King et al. was less trustworthy due to a very one-sided, positive evaluation of the intervention without discussion of the researcher's position. We had very minor concerns, because this limitation related only to one of eleven studies, the study contributed to this review finding to only a small extent and therefore, did not affect the review finding notably.	**No or very minor concerns**<br>The majority of underlying data directly and unambiguously supported the review finding and there were no contradictory data.	**No or very minor concerns**<br>Eleven studies contributed to this review finding and represented a variety of examples that illustrated or explained the finding.	**Minor concerns**<br>Although the phenomenon of interest in the majority of studies was of indirect relevance (experiences and descriptions instead of preferences and wishes), preferences and wishes were or could be derived from them directly and therefore, we did not have concerns that this negatively impacted the confidence in the review finding. However, in the studies of Modig et al., Tiilikainen et al. and Schulman-Green et al., parts of data could possibly not have been clearly differentiated from institutional care and the two latter contributed a notable part of data to the review finding.	**High**	Thirteen studies contributed to this review finding. There were no or very minor concerns regarding methodological limitations, coherence, adequacy and relevance. Although there were minor concerns regarding relevance, this did not impact the strength of the review finding.

Summary of review finding	Studies contributing to the review finding	Methodological limitations	Coherence	Adequacy	Relevance	Confidence in the evidence	Explanation of CERQual assessment
					Nevertheless, since these issues affected only three out of thirteen studies, we had minor concerns about the impact on confidence.		
**16. Older people expect healthcare professionals to be knowledgeable.**	[1, 4-6, 8-11, 13, 15, 16, 20]	**No or very minor concerns**<br><br>The study of King et al. was less trustworthy due to a very one-sided, positive evaluation of the intervention without discussion of the researcher's position. We had very minor concerns, because this limitation related only to one of twelve studies, the study contributed to this review finding to only a small extent and therefore, did not affect the review finding notably.	**No or very minor concerns**<br><br>The majority of underlying data directly and unambiguously supported the review finding and there were no contradictory data.	**No or very minor concerns**<br><br>Twelve studies contributed to this review finding and represented a variety of examples that illustrated or explained the finding.	**Moderate concerns**<br><br>Although the phenomenon of interest in the majority of studies was of indirect relevance (experiences and descriptions instead of preferences and wishes), preferences and wishes were or could be derived from them directly and therefore, we did not have concerns that this negatively impacted the confidence in the review finding. However, in five out of twelve studies, parts of data could possibly not have been clearly differentiated from institutional care or services not provided by healthcare professionals. Moreover, these five studies contributed a notable part of data to the review finding, so we had moderate concerns about the impact on confidence.	**High**	Twelve studies contributed to this review finding. There were no or very minor concerns regarding methodological limitations, coherence and adequacy. Although there were moderate concerns regarding relevance, the review finding was still a valid representation of the data.
**17. Older people value healthcare professionals'**	[5, 10-13, 15, 20]	**Moderate concerns**<br><br>The study of King et al. was less trustworthy due to a very	**No or very minor concerns**	**Minor concerns**<br><br>Seven studies contributed to this review finding and	**No or very minor concerns**	**Moderate**	Seven studies contributed to this review finding. There were no or very minor concerns regarding

Summary of review finding	Studies contributing to the review finding	Methodological limitations	Coherence	Adequacy	Relevance	Confidence in the evidence	Explanation of CERQual assessment
**communication skills.**		one-sided, positive evaluation of the intervention without discussion of the researcher's position. We had moderate concerns because this limitation related to one of seven studies and therefore, weakened the confidence in this review finding.	The majority of underlying data directly and unambiguously supported the review finding and there were no contradictory data.	represented a variety of examples and descriptions that supported the review finding. However, half of the underlying data were provided by only one study (King et al.).	Although the phenomenon of interest in the majority of studies was of indirect relevance (experiences and descriptions instead of preferences and wishes), preferences and wishes were or could be derived from them directly and therefore, we did not have concerns that this negatively impacted the confidence in the review finding. In two out of seven studies, parts of data could possibly not have been clearly differentiated from institutional care or services provided by healthcare professionals. However, these two studies contributed only a very small part of data to the review finding, so we had only very minor concerns about the impact on confidence.		coherence and relevance. However, there were minor concerns regarding adequacy and moderate concerns regarding methodological limitations. Altogether, we found that this weakened the review finding, but to a limited extent.
**18. Older people wish to receive personal attention.**	[1-3, 5-7, 9-14, 17, 19, 21, 22]	**No or very minor concerns**<br><br>The analysis of Bjornsdottir did not report on discrepant results and was conducted by only one researcher. The study of King et al. was less trustworthy due to a very one-sided, positive evaluation of the intervention without	**No or very minor concerns**<br><br>The vast majority of underlying data directly and unambiguously supported the review finding and there were no contradictory data.	**No or very minor concerns**<br><br>Sixteen studies contributed to this review finding and represented a variety of examples that illustrated or explained the finding.	**No or very minor concerns**<br><br>Although the phenomenon of interest in the majority of studies was of indirect relevance (experiences and descriptions instead of preferences and wishes),	**High**	Sixteen studies contributed to this review finding. There were no or very minor concerns regarding methodological limitations, coherence, adequacy and relevance.

Summary of review finding	Studies contributing to the review finding	Methodological limitations	Coherence	Adequacy	Relevance	Confidence in the evidence	Explanation of CERQual assessment
		discussion of the researcher's position. The study of Martin-Matthews et al. did not report on ethical approval, but the study's aim and results did not seem to be affected by that. We had very minor concerns, because these limitations related to only three of sixteen studies and therefore, did not affect the review finding notably.			preferences and wishes were or could be derived from them directly and therefore, we did not have concerns that this negatively impacted the confidence in the review finding. In the studies of Krothe et al., Soodeen et al., and Tiilikainen et al., parts of data could possibly not have been clearly differentiated from institutional care or services not provided by healthcare professionals. However, this was subject to only three out of sixteen studies and the three studies in question contributed only a very small part of data to the review finding.		
**19. Older people value close, long-term relationships.**	[1, 2, 5-7, 9-11, 14, 18, 21, 22]	**Minor concerns**<br><br>The analysis of Bjornsdottir did not report on discrepant results and was conducted by only one researcher. The study of King et al. was less trustworthy due to a very one-sided, positive evaluation of the intervention without discussion of the researcher's position. The study of Martin-Matthews et al. did not report on ethical approval, but the study's aim and results did not seem to be affected by	**No or very minor concerns**<br><br>The vast majority of underlying data directly and unambiguously supported the review finding and there were no contradictory data.	**No or very minor concerns**<br><br>Twelve studies contributed to this review finding and represented a variety of examples that illustrated or explained the finding.	**No or very minor concerns**<br><br>Although the phenomenon of interest in the majority of studies was of indirect relevance (experiences and descriptions instead of preferences and wishes), preferences and wishes were or could be derived from them directly and therefore, we did not have concerns that this negatively impacted the	**High**	Twelve studies contributed to this review finding. There were no or very minor concerns regarding coherence, adequacy and relevance. Although there were minor concerns regarding methodological limitations, this was only due to a small number of studies and there was no impact on the review finding in total.

Summary of review finding	Studies contributing to the review finding	Methodological limitations	Coherence	Adequacy	Relevance	Confidence in the evidence	Explanation of CERQual assessment
		that. We had minor concerns, because these limitations related to only three of thirteen studies and therefore, weakened the confidence in the review finding to a limited extent.			confidence in the review finding. In the studies of Krothe et al. and Soodeen et al., parts of data could possibly not have been clearly differentiated from services not provided by healthcare professionals. However, this was subject to only two out of twelve studies.		
**20. Older people want to be treated in a friendly way.**	[1-3, 5, 7, 9-11, 13, 17, 21]	**No or very minor concerns**<br>The analysis of Bjornsdottir did not report discrepant results and was conducted by only one researcher. The study of King et al. was less trustworthy due to a very one-sided, positive evaluation of the intervention without discussion of the researcher's position. We had very minor concerns, because these limitations related to only two of eleven studies, the studies contributed to this review finding only to a small extent and therefore, did not affect the review finding notably.	**No or very minor concerns**<br>The vast majority of underlying data directly and unambiguously supported the review finding and there were no contradictory data.	**No or very minor concerns**<br>Eleven studies contributed to this review finding and represented a variety of examples that illustrated or explained the finding.	**Minor concerns**<br>Although the phenomenon of interest in the majority of studies was of indirect relevance (experiences and descriptions instead of preferences and wishes), preferences and wishes were or could be derived from them directly and therefore, we did not have concerns that this negatively impacted the confidence in the review finding. However, in the studies of Soodeen et al., and Tiilikainen et al., parts of data could possibly not have been clearly differentiated from institutional care or services not provided by healthcare professionals and this may have affected the strength of the review finding.	**High**	Eleven studies contributed to this review finding. There were no or very minor concerns regarding methodological limitations, coherence and adequacy. Although there were minor concerns regarding relevance, this was only due to a small number of studies and there was no impact on the review finding in total.

Summary of review finding	Studies contributing to the review finding	Methodological limitations	Coherence	Adequacy	Relevance	Confidence in the evidence	Explanation of CERQual assessment
**21. Older people value open and confidential communication.**	[1, 5, 6, 8-15, 18, 21]	**No or very minor concerns**<br><br>The study of King et al. was less trustworthy due to a very one-sided, positive evaluation of the intervention without discussion of the researcher's position. We had very minor concerns, because this limitation related to only two of thirteen studies, the studies contributed to this review finding only to a very small extent and therefore, did not affect the review finding notably.	**No or very minor concerns**<br><br>The majority of underlying data directly and unambiguously supported the review finding and there were no contradictory data.	**No or very minor concerns**<br><br>Thirteen studies contributed to this review finding and represented a variety of examples that illustrated or explained the finding.	**No or very minor concerns**<br><br>Although the phenomenon of interest in the majority of studies was of indirect relevance (experiences and descriptions instead of preferences and wishes), preferences and wishes were or could be derived from them directly and therefore, we did not have concerns that this negatively impacted the confidence in the review finding. In the studies of Modig et al., Krothe and Soodeen et al., parts of data could possibly not have been clearly differentiated from institutional care or services not provided by healthcare professionals. However, this was subject to only three out of thirteen studies and the three studies in question contributed only a very small part of data to the review finding.	**High**	Thirteen studies contributed to this review finding. There were no or very minor concerns regarding methodological limitations, coherence, adequacy and relevance.
**22. Older people want to be involved in decisions and care.**	[1, 2, 4-6, 8, 9, 11, 12, 14-18, 21, 22]<br><br>Abweichend: [2, 4, 8, 16]	**Minor concerns**<br><br>The analysis of Bjornsdottir did not report on discrepant results and was conducted by only one researcher. The study of King et al. was less	**Minor concerns**<br><br>The majority of underlying data directly referred to the wish of being involved in decisions and care, but	**No or very minor concerns**<br><br>Sixteen studies contributed to this review finding and represented a variety of examples that	**Minor concerns**<br><br>Although the phenomenon of interest studied in the majority of studies was of indirect relevance (experiences	**Moderate**	Sixteen studies contributed to this review finding. There were no or minor concerns regarding adequacy, but minor concerns regarding methodological limitations,

Summary of review finding	Studies contributing to the review finding	Methodological limitations	Coherence	Adequacy	Relevance	Confidence in the evidence	Explanation of CERQual assessment
		trustworthy due to a very one-sided, positive evaluation of the intervention without discussion of the researcher's position. The study of Martin-Matthews et al. did not report on ethical approval, but the study's aim and results did not seem to be affected by that. We had minor concerns, because these limitations related to three of sixteen studies and therefore, weakened the confidence in the review finding to a limited extent.	within some of the studies, it was described that some older people wished to take a passive role. However, we described this as minor instead of moderate concern, because the conditions and variety of opinions in general were part of the review finding's description, and the ambiguous data clearly represented a minority.	illustrated or explained the finding.	and descriptions instead of preferences and wishes), preferences and wishes were or could be derived from them directly and therefore, we did not have concerns that this negatively impacted the confidence in the review finding. In the studies of Modig et al., Tiilikainen et al., Schulman-Green et al., Krothe and Soodeen et al., parts of data could possibly not have been clearly differentiated from services not provided by healthcare professionals or in institutional settings. However, this was subject to only four out of sixteen studies and the studies in question contributed only a relatively small part of data to the review finding, so we had minor concerns about the impact on confidence.		coherence and relevance. In total, we found that the review finding lost strength, in particular due to contradictory data.
**23. Older people value activity.**	[3, 4, 6, 9, 10, 12-15, 19, 20, 22]	**No or very minor concerns**<br><br>The study of Martin-Matthews et al. did not report on ethical approval, but the study's aim and results did not seem to be affected by that. We had very minor concerns because this limitation related to only one	**No or very minor concerns**<br><br>The majority of underlying data directly and unambiguously supported the review finding and there were no contradictory data.	**No or very minor concerns**<br><br>Twelve studies contributed to this review finding and represented a variety of examples that illustrated or explained the finding.	**No or very minor concerns**<br><br>Although the phenomenon of interest in the majority of studies was of indirect relevance (experiences and descriptions instead of preferences and wishes),	**High**	Twelve studies contributed to this review finding. There were no or very minor concerns regarding methodological limitations, coherence, adequacy and relevance.

**Summary of review finding**	**Studies contributing to the review finding**	**Methodological limitations**	**Coherence**	**Adequacy**	**Relevance**	**Confidence in the evidence**	**Explanation of CERQual assessment**
		of twelve studies, the study contributed to the review finding only to a very small extent and therefore, did not affect the review finding notably.			preferences and wishes were or could be derived from them directly and therefore, we did not have concerns that this negatively impacted the confidence in the review finding. In the studies of Walker et al. und Krothe, parts of data could possibly not have been clearly differentiated from institutional care or services not provided by healthcare professionals. However, this was subject to only two out of twelve studies and the two studies in question contributed only a very small part of data to the review finding.		

References

1. Berkelmans PG, Berendsen AJ, Verhaak PF, van der Meer K. Characteristics of general practice care: what do senior citizens value? A qualitative study. BMC Geriatr. 2010;10:80. https://doi.org/10.1186/1471-2318-10-80.

2. Bjornsdottir K. 'Holding on to life': An ethnographic study of living well at home in old age. Nurs Inq. 2018;25(2):1. https://doi.org/10.1111/nin.12228.

3. Faeo SE, Bruvik FK, Tranvag O, Husebo BS. Home-dwelling persons with dementia's perception on care support: Qualitative study. Nurs Ethics. 2020. https://doi.org/10.1177/0969733019893098.

4. Gowing A, Dickinson C, Gorman T, Robinson L, Duncan R. Patients' experiences of a multidisciplinary team-led community case management programme: a qualitative study. BMJ Open. 2016;6(9):e012019. https://doi.org/10.1136/bmjopen-2016-012019.

5. King AII, Boyd ML, Dagley L, Raphael DL. Implementation of a gerontology nurse specialist role in primary health care: Health professional and older adult perspectives. J Clin Nurs. 2018;27(3-4):807-18. https://doi.org/10.1111/jocn.14110.

6. Krothe JS. Constructions of elderly people's perceived needs for community-based long-term care. Indiana University School of Nursing; 1992.

7. Michel T, Helena Lenardt M, Hautsch Willig M, Maria Alvarez A. From real to ideal - the health (un)care of long-lived elders. Rev Bras Enferm. 2015;68(3):343-9. https://doi.org/10.1590/0034-7167.2015680304i.

8. Modig S, Kristensson J, Troein M, Brorsson A, Midlöv P. Frail elderly patients' experiences of information on medication. A qualitative study. BMC Geriatr. 2012;12(1):46. https://doi.org/10.1186/1471-2318-12-46.

9. Moe A, Hellzen O, Enmarker I. The meaning of receiving help from home nursing care. Nurs Ethics. 2013;20(7):737-47. https://doi.org/10.1177/0969733013478959.

10. Sandberg M, Jakobsson U, Midlov P, Kristensson J. Case management for frail older people - a qualitative study of receivers' and providers' experiences of a complex intervention. BMC Health Serv Res. 2014;14. https://doi.org/10.1186/1472-6963-14-14.

11. Soodeen RA, Gregory D, Bond JB. Home care for older couples: "It feels like a security blanket.. ". Qual Health Res. 2007;17(9):1245-55. https://doi.org/10.1177/1049732307307339.

12. Spoorenberg SLW, Wynia K, Fokkens AS, Slotman K, Kremer HPH, Reijneveld SA. Experiences of Community-Living Older Adults Receiving Integrated Care Based on the Chronic Care Model: A Qualitative Study. PLoS One. 2015;10(10):1. https://doi.org/10.1371/journal.pone.0137803.

13. Toien M, Bjork IT, Fagerstrom L. Older users' perspectives on the benefits of preventive home visits. Qual Health Res. 2015;25(5):700-12. https://doi.org/10.1177/1049732314553595.

14. Turjamaa R, Hartikainen S, Kangasniemi M, Pietila AM. Living longer at home: a qualitative study of older clients' and practical nurses' perceptions of home care. J Clin Nurs. 2014;23(21-22):3206-17. https://doi.org/10.1111/jocn.12569.

15. van Blijswijk SCE, de Waard CS, van Peet PG, Keizer D, von Faber M, de Waal MWM, et al. Wishes and needs of community-dwelling older persons concerning general practice: A qualitative study. PLoS One. 2018;13(7):14. https://doi.org/10.1371/journal.pone.0200614.

16. Schulman-Green DJ, Naik AD, Bradley EH, McCorkle R, Bogardus ST. Goal setting as a shared decision making strategy among clinicians and their older patients. Patient Educ Couns. 2006;63(1-2):145-51. https://doi.org/10.1016/j.pec.2005.09.010.

17. Tiilikainen E, Hujala A, Kannasoja S, Rissanen S, Närhi K. "They're always in a hurry" – Older people´s perceptions of access and recognition in health and social care services. Health Soc Care Community. 2019;27(4):1011-8. https://doi.org/10.1111/hsc.12718.

18. van Kempen JA, Robben SH, Zuidema SU, Rikkert MG, Melis RJ, Schers HJ. Home visits for frail older people: a qualitative study on the needs and preferences of frail older people and their informal caregivers. Br J Gen Pract. 2012;62(601):554-60. https://doi.org/10.3399/bjgp12X653606.

19. Behm L, Ivanoff SD, Ziden L. Preventive home visits and health: experiences among very old people. BMC Public Health. 2013;13:378. https://doi.org/10.1186/1471-2458-13-378.

20. Walker R, Ratcliffe J, White A, Visvanathan R. Dementia assessment services: What are the perceptions of older people? Australas J Ageing. 2018;37(1):43-7. https://doi.org/10.1111/ajag.12455.

21. Jarling A, Rydstrom I, Ernsth-Bravell M, Nystrom M, Dalheim-Englund AC. Becoming a guest in your own home: Home care in Sweden from the perspective of older people with multimorbidities. Int J Older People Nurs. 2018;13(3). https://doi.org/10.1111/opn.12194.

22. Martin-Matthews A, Sims-Gould J. Employers, home support workers and elderly clients: identifying key issues in delivery and receipt of home support. Healthc Q. 2008;11(4):69-75. https://doi.org/10.12927/hcq.2008.20073.
